# Secretome and Proteome of Extracellular Vesicles Provide Protein Markers of Lung and Colorectal Cancer

**DOI:** 10.3390/ijms26031016

**Published:** 2025-01-25

**Authors:** Natalia Soloveva, Svetlana Novikova, Tatiana Farafonova, Olga Tikhonova, Victor Zgoda

**Affiliations:** Laboratory of Systems Biology, Institute of Biomedical Chemistry, 119121 Moscow, Russia; n.solovyova@ibmc.msk.ru (N.S.); novikova@ibmc.msk.ru (S.N.); farafonova.tatiana@ibmc.msk.ru (T.F.); ovt@ibmh.msk.su (O.T.)

**Keywords:** extracellular vesicles, secretome, proteome, lung cancer, colorectal cancer

## Abstract

Colorectal cancer (CRC) and lung cancer (LC) are leading causes of cancer-related mortality, highlighting the need for minimally invasive diagnostic, prognostic, and predictive markers for these cancers. Proteins secreted by a tumor into the extracellular space directly, known as the tumor secretome, as well as proteins in the extra-cellular vesicles (EVs), represent an attractive source of biomarkers for CRC and LC. We performed proteomic analyses on secretome and EV samples from LC (A549, NCI-H23, NCI-H460) and CRC (Caco2, HCT116, HT-29) cell lines and targeted mass spectrometry on EVs from plasma samples of 20 patients with CRC and 19 healthy controls. A total of 782 proteins were identified across the CRC and LC secretome and EV samples. Of these, 22 and 44 protein markers were significantly elevated in the CRC and LC samples, respectively. Functional annotation revealed enrichment in proteins linked to metastasis and tumor progression for both cancer types. In EVs isolated from the plasma of patients with CRC, ITGB3, HSPA8, TUBA4A, and TLN1 were reduced, whereas FN1, SERPINA1, and CST3 were elevated, compared to healthy controls. These findings support the development of minimally invasive liquid biopsy methods for the detection, prognosis, and treatment monitoring of LC and CRC.

## 1. Introduction

According to the World Health Organization (WHO), in 2020, lung cancer (LC) and colorectal cancer (CRC) were two leading causes of cancer-related mortality. Such high mortality is associated with their late diagnosis. In the case of CRC, the tumor is detected in the late stages in 60% of patients and the 5-year survival rate of patients diagnosed with metastatic CRC is only 12.5% [1]. In addition, the CRC incidence in patients under 50 years of age has doubled over the past three decades [2]. In the case of LC, the disease is diagnosed in late stages in 84% of patients [3], and the 5-year survival rate for metastatic LC is only 4–16% [4].

The key to the successful treatment of both LC and CRC is the development and implementation of screening programs for early diagnostics. The early detection of CRC is especially important, since it can take from 10 to 15 years for a polyp to develop into a malignant tumor [5].

Currently, the most commonly used CRC screening tools include the fecal occult blood test (FOBT) and the fecal immunochemical test (FIT) followed by colonoscopy in the case of a positive result [6]. However, the FOBT does not detect precancerous intestinal lesions that have a lower tendency to bleed [7]. In addition, diagnostics using the FIT show a false-positive result in almost 50% of cases. As a result, colonoscopy combined with biopsy, which is associated with the risks of bleeding and intestinal perforation, is performed in healthy people [6]. The testing of antibodies to carcinoembryonic antigen (CEA) is the most widely used approach for the minimally invasive diagnosis of CRC in clinical practice. However, the method has limited sensitivity and specificity [8].

In the case of LC, the currently used screening diagnostics rely on instrumental methods, such as low-dose computed tomography followed by biopsy in the case of the detection of suspicious lesions in a patient with a high risk of developing LC, mainly due to a history of smoking [9]. As in the case of CRC, the main drawback of this method is the high percentage of false-positive results (27–50%) [10].

As shown by recent large-scale clinical trials (NLST, NELSON in the case of LC, SCREESCO in the case of CRC) [11,12,13], the current screening programs for the early diagnosis of LC and CRC have significantly improved the overall survival of patients. At the same time, the associated risks, the need to use expensive, complex equipment and the need for the involvement of qualified medical personnel to perform the study require further search for alternative markers of LC and CRC.

Like many types of cancer, LC and CRC are molecularly heterogeneous, and the molecular landscape of the tumor can determine different treatment strategies. In CRC, multiple genetic changes are detected, including mutation of the adenomatous polyposis coli (APC), mutation of the *K-ras* or *p53* genes, dysfunction of the DNA damage repair system, microsatellite instability (MSI), and chromosomal instability (CIN). The mutation status is associated with drug resistance, which develops in the vast majority of Patients wit CRC [14].

In the context of LC, mutations in the *EGFR*, *ALK*, *ROS-1*, *BRAF*, *NTRK*, *MET*, *RET*, *HER2*, and *KRAS* genes are predictive markers of sensitivity to the corresponding tyrosine kinase inhibitors (TKIs). In addition, the protein expression level of the programmed cell death receptor ligand 1 (PD-L1) is a predictive marker of the effectiveness of immunotherapy, namely the use of immune checkpoint inhibitors (ICIs) [15].

In the context of additional approaches to early diagnostics and prognosis/prediction of the disease outcome, the search for new markers of LC and CRC will optimize the therapy of these diseases.

It has been previously shown that tumors can actively secrete individual components (tumor cells, circulating tumor DNA (ctDNA), microRNA, proteins, and extracellular vesicles (EVs)) into the extracellular environment. The secreted components may finally reach the blood plasma, where they can be detected. This assumption underlies the concept of liquid biopsy as a promising minimally invasive diagnostic method. In the case of LC, ctDNA is already used to detect mutations in the EGFR gene with the subsequent personalization of therapy [9].

Among the components secreted by the tumor, the secretome, which represents a set of proteins directly secreted by tumor cells into the external environment, and proteins of the EVs are of particular interest. The secretome and EV proteins are directly associated with the tumor phenotype, and protein analytes are also characterized by higher stability and resistance to degradation compared to ctDNA and microRNA. The EV bilipid membrane provides additional protection from proteases and degradation. [16].

Despite the fact that plasma is traditionally used in clinical practice for minimally invasive diagnostics, it is an extremely complex material in terms of the detection of protein analytes, especially low-copy proteins. The reason for this is the so-called proteomic iceberg effect. This effect is based on the widest range of plasma proteome concentrations, covering 10 orders of magnitude [17], as well as a pronounced quantitative imbalance, in which a dozen protein analytes, the so-called highly abundant proteins (albumin, immunoglobulins, transthyretin, etc.), represent about 90% of the total plasma protein mass in the blood [18].

The proteomic iceberg effect can be reduced by using mass spectrometry (MS), characterized by specificity and sensitivity. Currently, using multiplex MS analysis in whole plasma, it is possible to identify a proteomic signature including the proteins A1AT, APOA1, HP, LRG1 and PON3; this set of proteins was used to distinguish plasma samples obtained from patients with CRC and healthy volunteers [19]. In the case of LC, using the method of targeted high-resolution MS (parallel reaction monitoring), it was possible to identify six proteins, FLNA, TUBA4A, GSTO1, PRDX6, and ARHGDIB, which quantitatively differed in the blood samples of LC patients as compared to those of healthy controls [20]. On the other hand, even such powerful technology as shotgun MS in the data-independent acquisition (SWATH) mode allows for registering only 200–300 proteins in plasma, which are mainly high and medium copy number proteins. At the same time, thousands of proteins can be qualitatively and quantitatively assessed in cells and tissues [21].

Cell lines are successfully used as LC and CRC models for MS-based searching for protein markers. In this work, we have used the culture media of LC (A549, NCI-H23, and NCI-H460) and CRC (Caco2, HCT116, and HT-29) cells as a model for studying secreted tumor markers. We have employed a specially developed approach for the simultaneous isolation of EVs and preparation of the secretome from the same culture medium sample. Using high-resolution shotgun MS profiling and label-free relative quantification, we were able to identify secreted markers of intestinal and pulmonary origin. In addition, we have performed a targeted mass spectrometric analysis of EVs isolated from plasma samples of patients with CRC and healthy volunteers using an expanded panel of the EV-associated proteins.

## 2. Results

### 2.1. MS Analysis Identified Secreted Proteins Associated with LC and CRC and Potentially Detectable in Plasma

**Figure 1:**
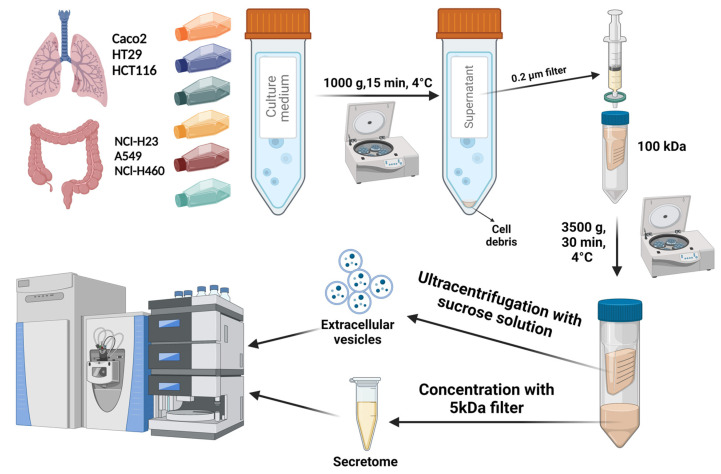
Experimental design of proteomic profiling of extracellular vesicles (EVs) and secretome samples derived from the model cell lines (Caco-2, HT29, HCT116 and A549, NCl-H23, NCl-H460). One initial volume of FBS-free cultural medium was used for obtaining EVs and secretome samples.

Using model cell lines as a source of secreted markers, we performed a proteomic analysis of samples obtained from one initial volume of FBS-free culture medium according to the experimental design shown in Figure 1.

**Figure 2:**
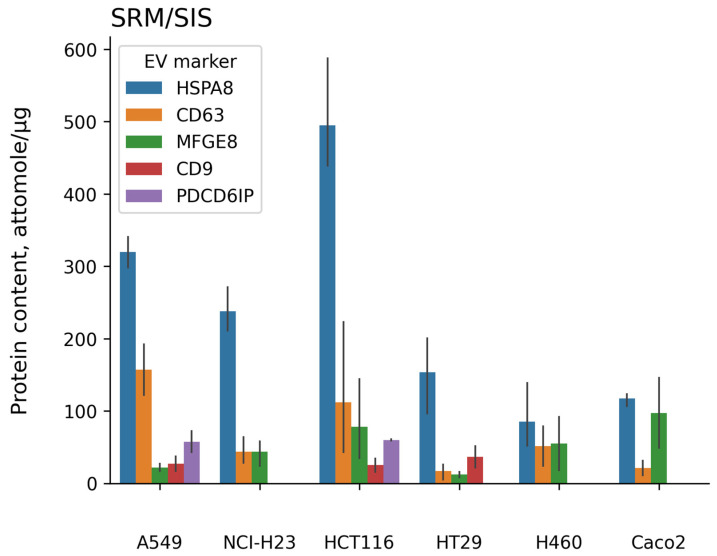
The result of measuring extracellular vesicle (EV) markers (HSPA8, CD63, MFGE8, CD9, and PDCD6IP) with the method of targeted mass spectrometry using isotope-labeled peptide standards (SRM/SIS) in samples of EVs isolated from the culture media of CRC cell lines (HCT116, HT29, and Caco2) and LC cell lines (A549, NCI-H23, and H460). The EVs were isolated in three replicates for each cell line studied, SRM/SIS measurements were performed in doubles for each EV sample.

To assess the effectiveness of EV isolation, we performed targeted mass-spectrometric measurement (SRM/SIS) of the convenient vesicle markers, i.e., HSPA8, CD63, MFGE8, CD9, and PDCD6IP, which are included in the Vesiclepedia Top100 list (https://microvesicles.org/extracellular_vesicle_markers, accessed on 25 November 2024) (Figure 2).

All measurements are listed in Supplementary Materials (Table S1, sheet ‘EV_cells_SRM_SIS Results’). The proteins HSPA8, CD63, and MFGE8 were detected in all cell lines studied in ranges of 52–587.8, 2–553, and 8–146 attomole/µg of total protein, respectively. HSPA8 was the most abundant EV marker for all cell lines studied, followed by CD63. CD9 protein was detected in EVs derived from the A549, HCT116, and HT29 cell lines at the levels of 27 ± 14, 25 ± 13, and 37 ± 21 attomole/µg of total protein, respectively. Programmed cell death 6-interacting protein (PDCD6IP) was measured at the levels of 58 ± 21 and 60 ± 2 attomole/µg of total protein in the EV samples that were related to A549 and HCT116 cell lines, respectively.

Shotgun MS analysis of the secretomes and proteomes of EVs isolated from the culture media of LC (A549, NCI-H23 and NCI-H460) and CRC (Caco2, HCT116 and HT-29) cells used as LC and CRC models resulted in the identification of 597 and 367 proteins, each with two unique peptides, respectively. A total of 182 proteins was common for both the secretome and proteome, while 415 and 185 proteins were detected only in the secretome and EV proteome samples, respectively (Figure S1).

Thus, a total of 782 proteins (identified by at least two unique peptides) secreted by tumor cells were detected. The full list of the secreted proteins is given in Supplementary Table S1. According to the biological annotation obtained in the GeneOntology database in the Cellular Localization category, 604 proteins (77.2%) belong to the first three most significant groups: GO:0070062~extracellular exosome, GO:0005576~extracellular region, and GO:0005615~extracellular space (Figure S2, Table S1, sheet ‘All detected proteins’).

The list of secreted proteins detected in our experiment in LC and CRC cells shares 88 proteins with the ProteinAtlas database proteins that were detected in plasma using immunochemical analysis (Figure S3). Moreover, 695 proteins from the experimentally detected secretome and EVs also coincide with proteins previously detected in plasma by MS methods and listed in ProteinAtlas (Figure S3).

Our previous studies have shown the possibility of detecting EV-associated proteins in cell models and in blood plasma [22,23]. In this work, the main goal was to expand the panel of potential secreted protein markers of LC and CRC.

### 2.2. Proteomic Composition of the Secretome Associated with LC and CRC

**Figure 3:**
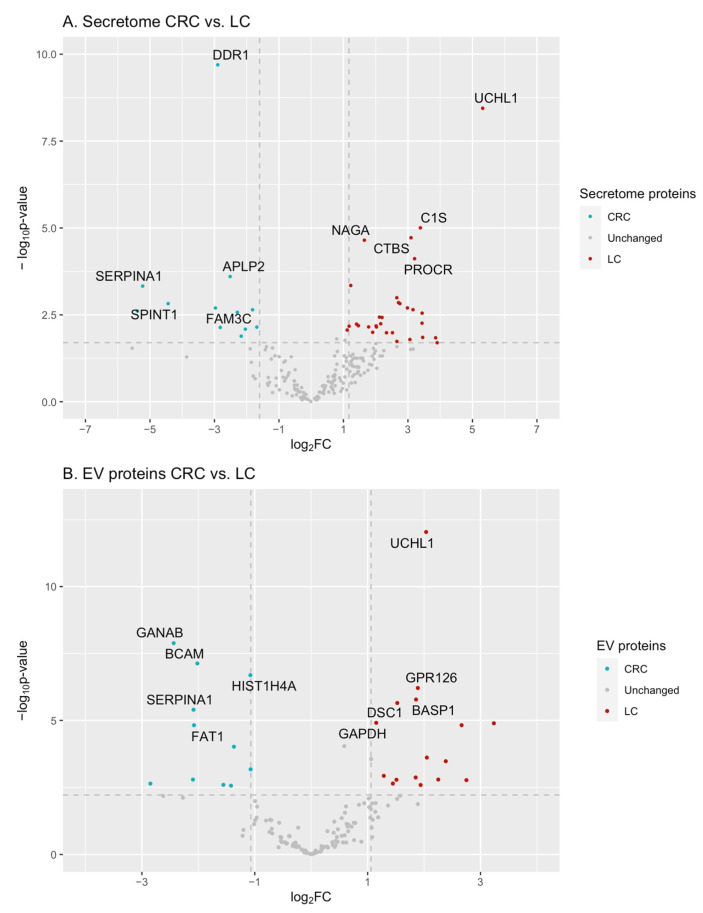
Volcano plots of proteins detected in the secretome (**A**) and EV (**B**) samples of LC and CRC cell lines. Proteins with significantly higher levels (FC > 2, FDR < 0.05) in the secretome samples of CRC cell lines (Caco-2, HT29, and HCT116) (left) and LC cell lines (A549, NCI-H23, and NCI-H460) (right). The names of 10 proteins with the most significant differences in levels between CRC- and LC-derived samples are shown.

In the secretome samples of all studied cell lines, 597 proteins were identified by at least two peptides. According to the results of the relative quantitative analysis, the contents of 42 proteins differed significantly (FC > 2, FDR < 0.05) in the secretome samples of intestinal and lung cell lines. The contents of 12 and 30 proteins were higher in the secretome samples of CRC cell lines (Caco-2, HT29, and HCT116) and LC cell lines (A549, NCI-H23, and NCI-H460), respectively (Figure 3A, Table S1, sheet ‘LC and CRC secreted markers’).

The five proteins that were most significantly upregulated in the secretome samples of CRC cell lines (Caco-2, HT29, and HCT116) were DDR1 (FC = 7.4), APLP2 (FC = 5.7), SERPINA1 (FC = 37.4), SPINT1 (FC = 21.6), and FAM3C (FC = 7.8) (listed in order of decreasing significance).

The five proteins UCHL1 (FC = 39.9), C1S (FC = 10.4), CTBS (FC = 8.5), NAGA (FC = 3.1), and PROCR (FC = 9.2) were the most significantly upregulated in the secretome samples of lung origin as compared to the secretome samples derived from CRC cell lines (listed in order of decreasing significance).

Principal component analysis (PCA) has shown that the set of proteins identified in this study can be used to distinguish between the LC and CRC secretome samples (Figure S4). However, the proteomic profile of the HCT116 cells from the CRC group was similar to that determined for the LC cells (Figures S4 and S5). This similarity is explained by the increased content of several proteins, such as ALCAM, APOA1BP, BTD, CD109, FAH, FDPS, FSCN1, LTA4H, PSAT1, RNPEP, TKT and TXNDC17 in HCT116 cells as compared to other CRC cell lines, such as Caco-2 and HT29 (Figures S4 and S5).

### 2.3. Proteomic Composition of LC and CRC-Associated EVs

In the EV samples of all studied lines, 367 proteins were identified by at least two peptides. The relative quantification revealed 29 proteins that differed significantly (FC > 2, FDR < 0.05) between the LC- and CRC-derived EV samples (Figure 3B, Figures S6 and S7). The abundance of 12 proteins (APOA1, APOE, BCAM, CST3, FAT1, GANAB, HIST1H4A, HIST2H3PS2, HSP90B1, KRT18, SERPINA1, and THBS1) was higher in the EV isolated from CRC cell lines (Caco-2, HT29, and HCT116) compared to the EV of lung origin (Table S1, sheet ‘LC and CRC secreted markers’). The abundance of 17 proteins (BASP1, CFH, CLEC11A, CLU, CNP, DSC1, GAPDH, GPR126, IGFBP2, IGFBP3, LTF, NAP1L1, P4HB, TIMP1, TKT, UCHL1, and VIM) was higher in the LC cell-derived EVs (A549, NCI-H23, and NCI-H460) as compared to the intestinal EVs (Table S1, sheet ‘LC and CRC secreted markers’).

The five proteins that were most significantly increased in the CRC cell line-derived EVs (Caco-2, HT29, and HCT116) were GANAB (FC = 5.4), BCAM (FC = 4.0), HIST1H4A (FC = 2.9), SERPINA1 (FC = 4.2), and FAT1 (FC = 4.2) (listed in order of decreasing significance). The top five proteins that were most significantly increased in the lung-derived EV samples (FC > 5) compared to the CRC cell line-derived EV proteome included UCHL1 (FC = 4.1), GPR126 (FC = 3.7), BASP1 (FC = 3.6), DSC1 (FC = 2.8), and GAPDH (FC = 2.2).

Among the secreted CRC markers, SERPINA1 was detected in both the secretome and EV samples. At the same time, UCHL1, TKT, and GPR126 proteins associated with LC were detected in the secretome samples and EV samples derived from LC cell lines.

A total of 23 secreted markers of CRC and 44 secreted markers of LC were identified (Table S1, sheet ‘LC and CRC secreted markers’). Of these, 19 secreted CRC markers (DDR1, HSP90AA1, SERPINA1, APLP2, RNASET2, APOA1, THBS1, PTPRF, HSP90B1, CST3, BCAM, KITLG, KRT18, SPINT1, PSAP, FAT1, FAM3C, APOE, and EZR) and 20 secreted LC markers (FDPS, IGFBP3, GSTP1, IGFBP2, AKR1B1, NAP1L1, CP, CLU, EFNA1, UCHL1, ALCAM, PSAT1, CTSL, CANX, FSCN1, PGK1, VIM, P4HB, TIMP1, and GAPDH) are involved in metastasis and tumor progression according to the DisGenNET database (Figure S8).

### 2.4. Targeted MS Analysis Using Stable Isotope Labels in EV Samples Isolated from the Blood of Patients with CRC and Healthy Volunteers

A panel of 32 EV-associated proteins was analyzed using the targeted MS method in the selected reaction monitoring (SRM) mode using the synthetic isotope-labeled peptide standards (SRM/SIS). We added SERPINA1 and CST3, identified as CRC markers in proteomic experiments on cell models, to the previously developed panel of EV-associated proteins. Measurements were performed in EVs isolated from the blood plasma samples of 20 patients with stage II CRC and 19 healthy volunteers. Information about patients is presented in Table 1. Detailed information on the SRM/SIS measurement is available in Table S1, sheet ‘EV HPL SRM_SIS Results’.

**Figure 4:**
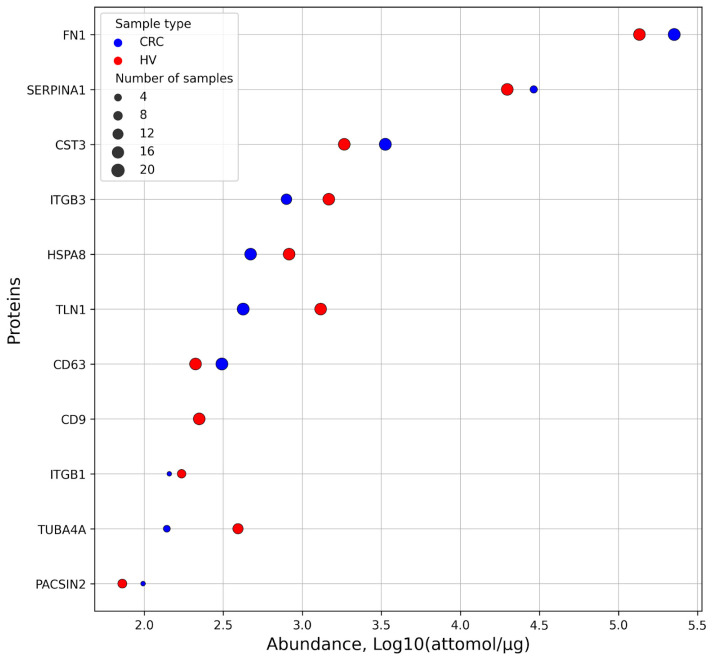
Quantitative data on 11 proteins detected in EV samples isolated from the blood plasma of patients with CRC and healthy volunteers (HVs). Average values expressed as attomole/mg total protein were log-transformed to base 10. Proteins measured in EV samples obtained from blood of patients with CRC and HVs are shown as blue and red dots, respectively. The size of the markers is proportional to the number of samples in which the proteins were detected.

The SRM/SIS analysis detected 11 of 32 target proteins in the EV samples isolated from the plasma of Patients wit CRC and healthy volunteers. These included: fibronectin (FN1), alpha-1-antitrypsin (SERPINA1), cystatin-C (CST3), integrin beta-3 (ITGB3), heat shock cognate 71 kDa protein (HSPA8), talin-1 (TLN1), CD63 antigen (CD63), CD9 antigen (CD9), integrin beta-1 (ITGB1), tubulin alpha-4A chain (TUBA4A), protein kinase C and casein kinase substrate in neurons protein 2 (PACSIN2) (Figure 4).

In EVs isolated from the plasma samples from healthy donors, conventional extracellular vesicle markers, i.e., HSPA8, CD9, and CD63, were detected at levels of 0.99 ± 0.56, 0.24 ± 0.1, and 0.23 ± 0.09 fmol/μg, respectively. Their measurements in the EVs isolated from the plasma of patients with patients CRC gave comparable data: 0.58 ± 0.45 fmol/μg for HSPA8, 0.24 ± 0.12 fmol/μg for CD9, and 0.15 ± 0.02 fmol/μg for CD63, respectively.

Given that the EVs were isolated from blood plasma, which is characterized by a wide dynamic range of protein concentrations, we evaluated the concentration range within the EV proteome. Additionally, we analyzed the frequency of CRC markers in the EV samples isolated from the blood plasma of patients with CRC and healthy donors. Figure 4 shows that the protein abundance varied by three and a half orders of magnitude. All proteins were detected in 10 or more EVs isolated from blood plasma. The highest level was revealed for FN1: 259 ± 123 fmol/μg in the EVs isolated from the plasma of patients with CRC and 150 ± 65 fmol/μg in the EVs isolated from the plasma of healthy volunteers, respectively. The FN1 levels were measured in all experimental samples. PACSIN2 protein was detected in only one sample of EVs isolated from patients with CRC and in half of the EV samples isolated from healthy volunteers; its lowest content in the EVs was 0.08 ± 0.02 fmol/μg. The CRC marker protein SERPINA1, detected in the cell models in the present study, was determined to be at a level of 31.2 ± 12.0 fmol/μg in all EVs isolated from patients with CRC and at a level of 22.1 ± 10.7 fmol/μg in all EV samples isolated from healthy volunteers. SERPINA1 was measured in all experimental samples. Another CRC marker protein, CST3, detected using cell models was measured in all EVs isolated from the blood samples of healthy volunteers at a level of 2.0 ± 0.9 fmol/μg. It was also registered in six EV samples obtained from patients with CRC at a level of 3.5 ± 0.8 fmol/μg total protein.

**Figure 5:**
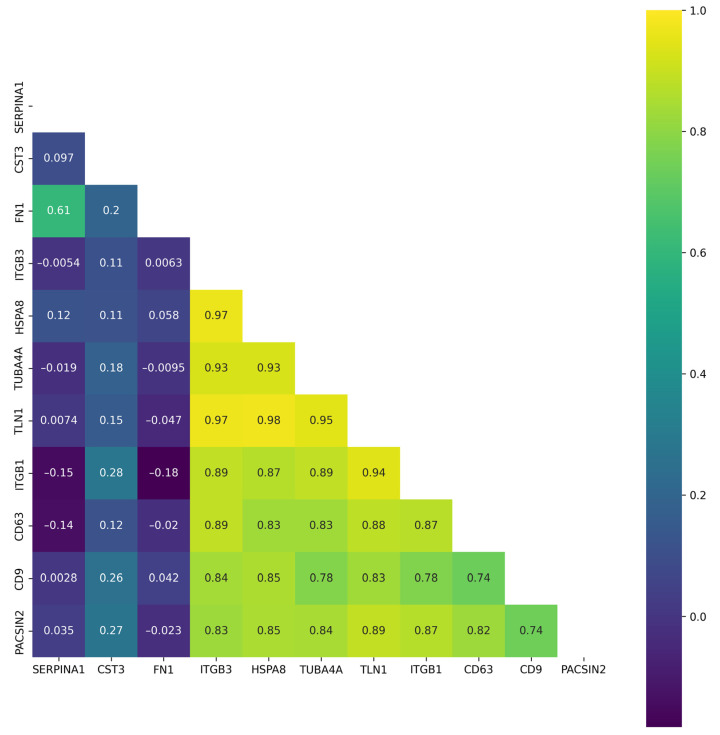
Correlation matrix for proteins detected in at least 10 EV samples isolated from blood plasma obtained from patients with CRC and healthy volunteers. The data for each EV sample are expressed as fmol/μg total protein, transformed using logarithm base 2.

Taking into account the fact that the expression of secreted proteins can be regulated by common mechanisms and that secreted proteins can form complexes, we constructed a correlation matrix for the protein profiles, shown in Figure 5.

Figure 5 shows that the highest correlation (r > 0.9) was observed for HSPA8, TUBA4A, and ITGB3 proteins. A moderate correlation (r > 0.7) was found for HSPA8, TUBA4A ITGB3, CD63, and CD9 proteins. The FN1, SERPINA1, and CST3 levels were inversely correlated with the abundance of other proteins found in the plasma-derived EVs.

Using SRM/SIS data for the proteins that were detected in at least 10 samples, a distance matrix was constructed showing the similarity between experimental samples (Figure S9).

## 3. Discussion

Model cell lines, as well as tumor tissues, are widely used to conduct MS-based searches for protein markers of LC and CRC. In such material, it is possible to successfully identify proteins that are significantly different in tumor cells/tissue compared to the control. However, the validation of potential protein markers in blood plasma, as with traditional clinical material used for minimally invasive diagnostics, often ends in failure.

In this study, we have focused on proteins potentially secreted by tumor cells into the extracellular space; this increases the chances of further detecting them in plasma. The analysis of EV-associated proteins in the whole human plasma could be hampered due to the dilution of vesicles and the interfering matrix effect of highly abundant proteins [17]. Applying precipitation with subsequent phenol-chloroform extraction for EV isolation from blood plasma, we obtained EV-enriched samples and removed highly abundant proteins and lipids. In this way, we improved the detection of extended EV-associated proteomes compared to the analysis of EV-associated proteins directly in blood plasma.

Compared to our previous study on LC and CRC cell line-derived EVs [24], we applied a modified method for vesicle isolation, which allowed us to obtain EV and secretome samples simultaneously from FBS-free cultural media. The secretome analysis enables the identification of additional proteins that are not detectable in the EVs alone (Figure S1). Moreover, the FBS-free approach to EV isolation enables the identification of EV-associated proteins that are annotated as being involved in growth factor binding, the regulation of RUNX3 expression and activity, and proteasome functioning according to the GO database. The data presented in the current study will help to expand the previously developed SRM panel [24] by incorporating new secreted proteins, providing a more comprehensive understanding of the molecular landscape of CRC and LC.

The list of secreted proteins of LC and CRC, registered in our experiment on cell models, significantly overlaps with the list of proteins that have ever been detected in plasma by the MS method, which is presented in the ProteinAtlas database (https://www.proteinatlas.org/, v. 23.0, accessed on 10 October 2024). This observation indicates the potential for detecting secreted proteins of pulmonary and intestinal origin in human blood plasma.

The EV and secretome samples were obtained from the same culture medium sample for each cell line studied. To a certain extent, the proteins identified in the secretome and EV samples overlapped between the two types of biomaterial. At the same time, the proteins detected only in the secretome samples (69.5% of all tumor cell secretome proteins) were biologically annotated as the proteins involved in metabolic processes, including the response to oxidative stress (Figure S10). The proteins detected in the EVs (50.4% of all EV tumor proteins) were involved in protein folding and telomere organization (Figure S10). Thus, the secretome and EVs of tumor cells represent functionally distinct protein repertoires. Moreover, the sets of proteins identified as markers of CRC and LC using semiquantitative mass spectrometry in different types of biomaterial are non-redundant, i.e., within each nosology they are defined either as part of the secretome or as part of the EVs. Thus, the data on the protein composition of the secretome and EVs are mutually supplementary.

In total, we were able to identify 23 and 44 secreted CRC and LC markers, respectively. According to the annotation of the secreted proteins against the categories of the DisGenNET database, many of them are involved in the processes of metastasis and tumor progression, and can therefore be used as prognostic markers of LC and CRC. Annotation of the secreted markers of LC in terms of their enrichment in signaling pathway components (online resource WebGestalt (https://www.webgestalt.org/, accessed on 10 October 2024)) showed that nine proteins (CNP, DSC1, FSCN1, GAPDH, IGFBP3, NAP1L1, P4HB, PGK1, and TKT) belonged to the VEGFA/VEGFR2 signaling pathway. Previously, in the context of LC, an association of mutations in the EGFR/KRAS genes and an increased expression of VEGFA/VEGFR2 in tumor tissue was demonstrated [25]. In addition, it has been shown that antiangiogenic therapy targeting the components of the VEGFA/VEGFR2 pathway was effective in the treatment of lung adenocarcinoma (LAC), but biomarkers are required to identify the target group of patients. Thus, the secreted LC markers identified in this work can be used to personalize therapy [26].

Among the proteins that distinguish between the secretome samples derived from the LC and CRC cell lines, epithelial discoidin domain-containing receptor 1 (DDR1) is especially interesting. In the secretome samples of intestinal origin, the DDR1 content was particularly different (FC = 7.4) compared to the samples of pulmonary origin. The DDR1 collagen receptor, the content of which was increased in the CRC secretome samples, has protein kinase activity; it is one of the main components of the extracellular matrix, is involved in the regulation of cell adhesion, and promotes the invasion of tumor and stem cells in a collagen-rich microenvironment [27]. Previously, using the immunohistochemical method, it was shown that the DDR1 content significantly increased in the tissue of highly malignant tumors; the latter suggests its putative role as a prognostic marker or therapeutic target [28]. In support of this hypothesis, it has been demonstrated that the DDR1 protein acts as an effective target for CRC therapy with targeted drugs such as the tyrosine kinase inhibitor nilotinb and monoclonal antibodies [29,30].

On the other hand, the most pronounced relative changes in the content of ubiquitin C-terminal hydrolase L1 (UCHL1) were noted in both the LC secretome (FC = 39.9) and LC EVs (UCHL1 (FC = 4.1) as compared to the CRC secretome and EVs. This enzyme is involved in the deubiquitination and degradation of damaged and misfolded proteins via the ubiquitin-proteasome system. Extensive studies of the functions of UCHL1 have shown that the increased expression of this protein contributes to the progression and metastasis of many types of cancer, including gastric cancer [31], breast cancer [32], and lung cancer [33]. In the case of LC, increased UCHL1 expression in airway epithelial cells was detected not only in patients with a confirmed diagnosis, but also in chronic smokers without signs of malignancy. Compared with non-smokers, the content of this protein demonstrated almost a 20-fold increase, suggesting a special role of UCHL1 in the early stages of tumor transformation of the epithelium [34]. A study conducted using the immunohistochemistry method on LC tissue samples showed increased expression of the protein compared to normal lung tissue [35]. It was shown that the level of UCHL1 expression determined in tumor tissue by the immunohistochemical method was associated with an unfavorable outcome in patients with LC and breast cancer, and that this protein could serve as a target for targeted therapy [33].

Besides UCHL1, it is especially worth mentioning the adhesion G protein-coupled receptor F5 GPR126 (FC = 3.7), according to the ProteinAtlas database (https://www.proteinatlas.org/, v. 23.0, accessed on 10 October 2024), is expressed predominantly in human respiratory system cells, including LC cell lines. At the same time, studies of the role of GPR126 in the context of gastric cancer and breast cancer have shown its involvement in the development of the tumor process [36,37].

The semiquantitative proteomic analysis of EVs resulted in the identification of proteins that differed between the EVs derived from LC and CRC cells. In the EVs of intestinal origin, the expression of the neutral alpha-glucosidase AB (GANAB) protein (FC = 7.4) differed most significantly compared to the EVs of pulmonary origin. In this context, there are just a few reports on the involvement of GANAB, particularly in the biology of CRC. At the same time, GANAB is a key enzyme regulating glycosylation, which, in turn, plays an important role in the occurrence and development of various tumors in general [38,39].

The proteomic analysis of three LC cell lines and three CRC cell lines at the level of the secretome and EVs revealed certain differences between the two tumor types. First of all, we have identified a specific pattern of protein expression for the HCT116 CRC cell line, both in the secretome and in the EVs. This HCT116-associated expression pattern was characterized by the increased expression of ALCAM, APOA1BP, BTD, CD109, FAH, FDPS, FSCN1, LTA4H, PSAT1, RNPEP, TKT, and TXNDC17 proteins as compared to the HT29 and Caco-2 CPP cell lines. Previously, in a meta-analysis of previously published data, it was shown that activated leukocyte cell adhesion molecule (ALCAM) was associated with the development and progression of CRC, and its increased expression was correlated with an unfavorable prognosis of the disease [40]. The proteins phosphoserine aminotransferase 1 (PSAT1) and transketolase (TKT) are involved in metabolic reprogramming in CRC [41,42]. Moreover, their increased expression, determined in tumor tissue by immunohistochemistry, was correlated with an unfavorable prognosis [43]. It is possible that the specific expression pattern of the HCT116 line is due to the presence of microsatellite instability (MSI) or a mutation in the KRAS gene (G13D) in these cells. In this context, it should be noted that that this mutation occurs in 30–50% of patients with CRC, and its presence determines their sensitivity to anti-EGFR inhibitors [44]. It is possible that assessment of the level of proteins representing the HCT116-associated expression pattern will help in predicting the outcome of the disease, and in personalizing CRC therapy.

In proteomic studies of EVs, it is important to evaluate possible artifacts that arise during their isolation. For example, in the case of using cell lines as model tumor objects, fetal bovine serum (FBS) is often added to the culture medium. During the subsequent MS analysis of EVs isolated from culture media containing FBS, despite strong dilution of the FBS by the medium, the serum proteins can be identified as human due to their high interspecies homology. In our work, the LC and CRC cells were specifically cultured in the absence of FBS, for 24 h before EV isolation, to reduce the risk of contamination with highly abundant serum proteins (albumin, immunoglobulins, etc.). Among the secreted CRC proteins, APOA1 (seven-fold more abundant in CRC EVs than in LC EVs) and APOE (six-fold more abundant in CRC EVs than in LC EVs) were detected; they were also found in plasma chylomicrons.

It is interesting that small intestinal cells are one of the main sites of APOA1 synthesis [45]. In addition, it was previously shown that APOA1 had diagnostic value in CRC [46]. Another secreted marker of CRC EVs, apolipoprotein E (APOE), is found in EVs of neuronal origin [47,48]. In the context of CRC, APOE and its receptor LRP1 have been shown to be involved in metastasis, and increased APOE expression is associated with poor prognosis [49].

Some markers identified in this work as secreted proteins (e.g., DDR1 and UCHL1) have been positioned as tissue markers in studies by other groups, and the protein expression level of these markers was previously determined directly in tumor samples by immunohistochemistry based on the antigen–antibody interaction [50,51]. Although this approach is widely used in clinical practice, it is not applicable for the multiplexed analysis and simultaneous quantitative assessment of dozens of protein analytes. In this work, we used a targeted SRM/SIS mass spectrometric method to analyze EV-associated proteins in EVs isolated from the plasma of patients with CRC and healthy volunteers. Using this approach, we have determined there to be reduced levels of ITGB3, HSPA8, TUBA4A, and TLN1, as well as increased levels of FN1, SERPINA1, and CST3, in EVs obtained from patients as compared to healthy controls. SERPINA1 and CST3 proteins were added to the previously developed panel of EV-associated markers based on the results obtained in the model lines described in this work.

In our study, alpha-1-antitrypsin (SERPINA1) was detected in EVs and in the secretome derived from CRC cells, and it was the only redundant CRC marker detected in both types of secreted biomaterial. This result is consistent with the detection of SERPINA1 in both EVs and in the secretome samples derived from mesenchymal stem/stromal cells (MSCs) [52]. In addition, using shotgun MS, we previously determined there to be an increased content of SERPINA1 in EVs isolated from blood samples of patients with advanced CRC as compared to healthy volunteers [53].

We applied mass spectrometric analysis, which allowed us to identify secreted proteins of pulmonary and intestinal origin that could potentially be detected in blood plasma. Additionally, a meta-analysis of the data on organotypic profiles in blood plasma obtained by alternative methods, such as SomaLogic aptamers, [54] could be used to further expand the MRM panel.

## 4. Materials and Methods

### 4.1. Cultivation of Lung Cancer (A549, NCI-H23, and NCI-H460) and Colorectal Cancer (Caco2, HCT116, and HT-29) Cells

The LC cell lines A549, NCI-H23, and NCI-H460, and CRC cell lines HT29, HCT-116, and CaCo-2 were obtained from the Cell Culture Bank of the Institute of Biomedical Chemistry (IBMC) (Moscow, Russia). The cell lines features are presented in Table S2.

The cell lines were cultured in an atmosphere of 5% CO_2_ at 37 °C in DMEM/F-12 medium without glutamine (PanEco, Moscow, Russia) and supplemented with 10% fetal bovine serum (FBS) (for culturing Caco-2 cells, the FBS content was 20%) (Thermo Fisher Scientific, Waltham, MA, USA), 1% GlutaMAX (Thermo Fisher Scientific, Waltham, MA, USA), 1% essential amino acids (Thermo Fisher Scientific, Waltham, MA, USA), and 1% antifungal antibiotics (amphotericin B 0.25 g/mL, penicillin G 100 U/mL, streptomycin 100 g/mL) until 70–80% confluence was achieved. The cells were then washed twice with phosphate-buffered saline (PBS), and the culture medium was replaced with a medium without FBS. For further analysis, the culture medium was collected after 24 h. All cell lines were tested for mycoplasma contamination.

### 4.2. Simultaneous Isolation of Extracellular Vesicles and Secretome from Culture Fluid

Extracellular vesicles and secretome samples were obtained simultaneously using the same volume of the culture medium. For this purpose, an ultrafiltration and ultracentrifugation approach employing a sucrose cushion was used.

The culture medium (15 mL) was centrifuged at 1000× *g* for 15 min at 4 °C using an Allegra X-15R Centrifuge (Beckman Coulter, Indianapolis, IN, USA) in an SX4750A Swinging Bucket Rotor (Beckman Coulter, Indianapolis, IN, USA) to sediment cellular debris. The supernatant was then passed through a filter with a pore size of 0.2 μm (Merck Millipore Limited, Tullagree, Ireland). The resulting volume was then concentrated using 100 kDa cutoff centrifuge filters (Amicon, 100K, Merck Millipore Limited, Tullagree, Ireland) by centrifugation at 3500× *g* for 30 min at 4 °C (Allegra X-15R Centrifuge using the SX4750A Swinging Bucket Rotor, Beckman Coulter, Indianapolis, IN, USA) to a volume of 500 μL. The filtrate was then used to obtain the secretome, and the fraction on the filter was used to obtain the EVs.

### 4.3. Isolation of Extracellular Vesicles from the Culture Fluid

The fraction on the filter was centrifuged at 100,000× *g* for 90 min at 4 °C in an Optima MAX-XP Ultracentrifuge using a TLA-55 rotor (Beckman Coulter, Indianapolis, IN, USA). The resulting sediment was dissolved in 50 μL of 0.015% sodium cholate in 0.1 M PBS (pH 7.4) and layered on 26% sucrose solution in potassium phosphate buffer (r = 1.1082 g/mL) followed by ultracentrifugation at 120,000× *g* for 120 min at 4 °C.

The sucrose solution was collected, and the resulting precipitate was dissolved in a buffer containing 1% SDS in 100 mM Tris-HCl (pH 7.4) and sonicated using a Bandelin Sonopuls ultrasonic disintegrator with a probe (Bandelin Electronic GmbH & Co. KG, Berlin, Germany) at 30% power for 1 min on ice. The solution was then centrifuged for 20 min at 14,000× *g* and 20 °C. The total protein concentration in the obtained samples was determined colorimetrically using a commercial Pierce™ BCA Protein Assay Kit (Pierce, Rockford, IL, USA) in accordance with the manufacturer’s recommendations.

### 4.4. Secretome Preparation from the Culture Liquid

The filtrate obtained after centrifugation of the culture liquid through filters with a 100 kDa cutoff (Amicon, 100K, Merck Millipore Limited, Tullagree, Ireland) was concentrated using centrifugal filters with a 5 kDa cutoff (Agilent Technologies, Santa Clara, CA, USA) at 3500× *g* for 30 min at 4 °C (Allegra X-15R Centrifuge using the SX4750A Swinging Bucket Rotor, Beckman Coulter, Indianapolis, IN, USA). The resulting concentrate was dried on a rotary evaporator and dissolved in a buffer containing 100 mM Tris-HCl (pH 7.4) for protein extraction with methanol-chloroform as described previously [55]. The precipitate was dissolved in a buffer containing 1% SDS in 100 mM Tris-HCl (pH 7.4), treated using a Bandelin Sonopuls ultrasonic homogenizer (Bandelin Electronic GmbH & Co. KG, Berlin, Germany) (power 30% for 30 s on ice), and then centrifuged at 14,000× *g* for 10 min at 20 °C.

The concentration of total protein in the obtained samples was determined colorimetrically using a commercial Pierce™ BCA Protein Assay Kit (Pierce, Rockford, IL, USA) according to the manufacturer’s recommendations.

### 4.5. Enzymatic Digestion of Proteins from Secretome Samples

The enzymatic digestion of secretome samples was performed using the FASP (filter-aided sample preparation) protocol [56] with minor modifications. Samples were loaded onto 10 kDa cutoff concentrating filters by centrifugation at 11,000× *g* for 15 min at 20 °C. For reduction and alkylation of disulfide bonds, tris(2-carboxyethyl)phosphine (TCEP) and 2-chloroacetamide (CAA) were added to each sample to final concentrations of 30 mM and 50 mM, respectively, and incubated for 40 min at 80 °C. After addition of a buffer containing 8 M urea in 0.1 M Tris-HCl (pH 8.5) and centrifuged at 11,000× *g* for 15 min at 20 °C, the procedure was repeated 5 times. Then, the samples were washed three times with a buffer containing 50 mM triethylammonium bicarbonate (TEAB) (pH 8.5) by centrifugation as above. After addition of 50 μL of 50 mM TEAB, pH 8.5, 0.5 μg/μL trypsin solution (at a trypsin–protein mass ratio of 1:50, Promega, Fitchburg, WI, USA), samples were incubated overnight at 37 °C. Peptides were eluted by centrifugation at 11,000× *g* for 15 min at 20 °C and the filter was washed twice with 100 μL of 0.1% formic acid. The resulting samples were analyzed for peptide concentration by colorimetric analysis using the commercial Pierce™ BCA Protein Assay Kit (Pierce, Rockford, IL, USA) according to the manufacturer’s recommendations.

Peptides were dried and dissolved to a final concentration of 2 μg/μL in 0.1% formic acid.

### 4.6. Isolation of Extracellular Vesicles from Plasma Samples

Plasma samples from 20 patients with CRC and 19 healthy volunteers were obtained from the IBMC Biobank. Patients with a family predisposition to CRC were not included.The description of the patients is given in Table 1.

Venous blood from patients with CRC and healthy donors was collected in identical vacuum tubes with K_2_ EDTA. Plasma was obtained by centrifugation of whole blood at 1300× *g* for 10 min immediately after sampling. Hemolysis was assessed by visual inspection.

The resultant plasma was aliquoted, and samples were stored at −80 °C until analysis.

EVs were isolated from 30 µL plasma of each sample using a previously described protocol [22] and a commercial Total Exosome Isolation kit (Invitrogen, Thermo Fisher Scientific, Vilnus, Lithuania) in accordance with the manufacturer’s recommendations.

The resulting sediment was dissolved in 100 μL of 0.1 M Tris-HCl (pH 8.5) and methanol-chloroform extraction of the protein was performed. Briefly, 400 μL of 100% methanol (J.T. Baker, Avantor, Poland), 100 μL of chloroform (Sigma-Aldrich, St. Louis, MO, USA), and 300 μL of deionized water were added to the samples, and the mixture was thoroughly mixed after adding each component and then centrifuged at 14,000× *g* for 2 min at room temperature. The supernatant was collected and, after addition of 400 μL of 100% methanol, it was centrifuged again under the same conditions for 3 min. The resulting sediments were dissolved in 100 μL of 50 mM TEAB for subsequent proteomic analysis. The concentration of total protein in the samples was determined using a commercial Pierce™ BCA Protein Assay Kit (Pierce, Rockford, IL, USA) in accordance with the manufacturer’s recommendations.

### 4.7. Hydrolysis of EVs on S-Trap Spin Columns

Hydrolytic cleavage of EVs was performed using a S-trap spin column-based protocol (Protifi, Fairport, NY, USA) according to the manufacturer’s recommendations.

Ten percent SDS in 100 mM TEAB was added to each sample containing 100 μg protein so that the final concentrations were 5% SDS and 50 mM TEAB (pH 8.5). The resulting samples were treated using a Bandelin Sonopuls ultrasonic homogenizer (Bandelin Electronic GmbH & Co. KG, Berlin, Germany) (power 30% for 30 s on ice) and then the samples were centrifuged at 14,000× *g* for 10 min at 20 °C.

For alkylation and reduction of disulfide bonds, a solution containing 400 mM chloroacetamide (CAA, Sigma-Aldrich, St. Louis, MO, USA) and 500 mM tris(2-carboxyethyl)phosphine (Tris TCEP, Thermo Fisher Scientific, Waltham, MA, USA) was added to the samples to final concentrations of these ingredients of 30 mM and 50 mM, respectively. Samples were incubated at 80 °C for 40 min and then 12% phosphoric acid was added to the samples to a final concentration of 1.2%; samples were then mixed thoroughly and six parts of buffer (90% methanol in 100 mM TEAB (pH 8.5)) were added. Next, the samples were loaded to S-trap micro-columns in 175 μL and centrifuged at 4000× *g* for 1 min at 20 °C. The procedure was repeated until the sample was completely loaded, then the columns were washed four times with the same solution under the same conditions.

Protein digestion was performed using 0.2 ng/μL trypsin solution (Promega, Fitchburg, WI, USA), which was added to the samples at a trypsin–total protein ratio of 1:50. Samples were incubated for 2 h at 47 °C. Then, the peptides were eluted: 40 μL of a solution containing 0.2% formic acid in 50 mM TEAB (pH 8.5) was added and samples were centrifuged at 4000× *g* for 1 min at 20 °C. Next, 35 μL of a solution containing 0.2% formic acid in 50% acetonitrile was added and the samples were centrifuged again under the same conditions. The supernatant was dried in a vacuum concentrator (Concentrator 5301, Eppendorf, Berlin, Germany).

For proteomic profiling of LC and CRC cell-derived EVs, whole sample volume was subjected to tryptic digestion and total amount of tryptic peptides was measured by colorimetric analysis using the commercial Pierce^TM^ Quantitative Colorimetric Peptide Assay (Pierce^TM^ BCA Protein Assay Kit) according to the manufacturer’s recommendations. Peptides were dried and dissolved to a final concentration of 2 μg/μL in 0.1% formic acid. An equal injection volume was subjected to LC/MS-MS analysis. Before SRM analysis, the cell-derived EVs were spiked with SIS peptides. The final content of each SIS–peptide ratio was 40 fmole per µg of total tryptic peptides.

For SRM analysis of HPL-derived EVs, an equal quantity of total protein of 100 μg was subjected to tryptic digestion and total amount of tryptic peptides was measured by colorimetric analysis using the commercial Pierce^TM^ Quantitative Colorimetric Peptide Assay (Pierce^TM^ BCA Protein Assay Kit) according to the manufacturer’s recommendations. An equal injection volume was subjected to SRM analysis. Before analysis, the samples were dried in a vacuum concentrator and re-dissolved in 0.1% formic acid containing SIS peptides at an equimolar concentration of 500 fmol/µL. The final concentration of total tryptic peptides was 2 μg/μL. The final content of each SIS peptide was 28 fmole per µg of total tryptic peptides.

### 4.8. Shotgun Mass Spectrometric Analysis

Each sample was analyzed in three technical replicates. The peptide mixture was loaded onto a Zorbax 300SB-C18 enrichment column (particle diameter 5 μm, 5 mm × 0.3 mm) (Agilent Technologies, Santa Clara, CA, USA) and washed with mobile phase C (5% acetonitrile in 0.1% formic acid and 0. 05% trifluoroacetic acid) for 5 min at a flow rate of 3 µL/min. Peptides were separated on a Zorbax 300SB-C18 analytical column (particle diameter 3.5 μm, 150 mm × 75 μm) in the gradient of mobile phase B (80% acetonitrile solution in 0.1% formic acid) at a flow rate of 0.3 μL/min. The following parameters of the acetonitrile were used: the analytical column was washed with 2% mobile phase B for 3 min, then the concentration of mobile phase B was linearly increased to 40% for 67 min. Then, within 2 min, the concentration of mobile phase B was increased to 100%, and the analytical column was washed for 9 min with 100% mobile phase B. Next, the concentration of mobile phase B was reduced to 2% over 2 min, and the analytical column was equilibrated with 2% mobile phase B for 7 min.

MS analysis was carried out using a Q Exactive™ HF Hybrid Quadrupole-Orbitrap™ mass spectrometer (Thermo Fisher Scientific, Waltham, MA, USA) equipped with an Orbitrap mass analyzer. The maximum accumulation time for 3 × 10^6^ ions to obtain a MS scan with a resolution of 60,000 (*m*/*z* = 400) in the range of *m*/*z* = 380–1650 in the positive ionization mode was 25 ms. The 20 most intense ions recorded in the MS scan were selected for subsequent fragmentation provided that the AGC target value was greater than 10^4^. The HCD fragmentation type with normalized collision energy of 28% was used. Dynamic exclusion from tandem analysis was used: the exclusion duration was 60 s. The maximum accumulation time for 2 × 10^5^ ions to obtain a MS/MS scan with a resolution of 15,000 (for *m*/*z* = 400) in the positive ionization mode was 150 ms.

For identification mass spectrometry, data were loaded into the MaxQuant software (version 2.0.3.0, Max Planck Institute of Biochemistry, Martinsried, Germany). MS/MS spectra were identified using the built-in Andromeda algorithm against the FASTA file (UP000005640, UniProt Release 2022_02, 20,598 proteins, EMBL-EBI, Hinxton Cambridge, UK) and its inverted counterpart, alongside a built-in database of potential contaminants, to calculate the frequency of false positive identifications (FDR). The carbamidomethylation of cysteine was applied as a fixed modification, and methionine oxidation and N-terminal acetylation were used for variable modification. Trypsin was selected as the protease, and two missed cleavages were allowed. The tolerance for the precursor and fragment ions was set to 20 ppm. For proteins and peptides, the FDR threshold value was set to 0.01.

For quantitative analysis based on parent ion intensity, potential contaminants, false positive identifications, and proteins identified only by peptides that contained variable modifications were removed from the proteomic data. The statistical analysis was carried out using the Perseus 1.6.0.7 software (Max Planck Institute of Biochemistry, Martinsried, Germany). Proteins with more than 30% missing values in total across all samples were omitted from further analysis. Two-sample *t*-tests were used to assess differences between the two groups. The data were corrected for multiple comparisons using permutation-based FDR with a threshold value of 0.05, S0 = 0.1. Only the proteins identified by at least 2 unique peptides were used for quantification.

### 4.9. Targeted Proteomic Analysis in the Selected Reaction Monitoring (SRM) Mode

Before analysis, the samples were dried in a vacuum concentrator and re-dissolved in 0.1% formic acid containing SIS peptides at an equimolar concentration of 500 fmol/µL. The final contents of each SIS peptide were 40 fmol/µg and 28 fmol/µg of total protein for the cell line-derived and blood plasma-derived EV samples, respectively.

Chromatographic separation of peptides was performed using an Agilent 1200 series system (Agilent Technologies, Santa Clara, CA, USA) coupled to a TSQ Quantiva triple quadrupole mass analyzer (Thermo Fisher Scientific, Waltham, MA, USA). The sample was separated on a Zorbax 300SB-C18 analytical column (particle diameter 5 µm, 150 × 0.5 mm) (Agilent Technologies, Santa Clara, CA, USA) in an acetonitrile gradient at a flow rate of 20 µL/min. First, the column was equilibrated with 5% solution B (80% acetonitrile in 0.1% formic acid) and 95% solution A (0.1% formic acid) for 5 min. Then the concentration of solution B was linearly increased to 50% for 30 min, after which it was brought to 99% for 1 min. The column was washed with 99% solution B for 5 min; the concentration was returned to the initial conditions for 1 min, and then the column was equilibrated for 9 min.

Targeted mass spectrometric analysis was performed on a TSQ Quantiva triple quadrupole mass spectrometer (Thermo Fisher Scientific, Waltham, MA, USA) in the dynamic selected reaction monitoring (dSRM) mode with the following MS detector settings: capillary voltage of 4 kV, drying gas (nitrogen) flow rate of 7 l/min, axillary gas (nitrogen) flow rate of 5 l/min, capillary temperature of 350 °C, isolation window for the first and third quadrupoles of 0.7 Da, scan cycle time of 1.2 s, and gas pressure (argon) in the collision cell set at 1.5 mTorr. The retention time window on the reversed-phase column was 2.2 min for each precursor ion. The transitions given in Table S1 (sheets ‘SRM_table_EV_cells’ and ‘SRM_table_EV_HPL’) were used. The data were loaded into Skyline software version 4.1.0 (MacCoss Lab Software, Seattle, WA, USA), where SRM spectra were evaluated manually. The ratio of natural peptides to their SIS analogues was automatically calculated for each analyte.

For SIS peptides for which their natural counterparts were detected in blood plasma-derived EV samples, calibration curves were built (Figures S11–S13). The SIS peptides were measured at the levels of 75, 150, 300, 600,1200, 2400, and 4800 attomole, diluted in the presence of non-targeted peptide matrix. The LOD and LOQ were determined based on the slope of the calibration curves and 3 and 10 standard deviations of the response, respectively.

### 4.10. Bioinformatics Analysis and Data Visualization

Data annotation in terms of cellular localization and signaling pathways was performed using the WebGestalt online resource (https://www.webgestalt.org/, accessed on 10 October 2024) against the GO database categories “geneontology_Biological_Process_noRedundant” (FDR Method: BH).

Enrichment analysis was performed using the gseapy tool (v. 1.0.4) in python3 (v. 3.9.7) against the “DisGenNET”, “GO_Biological_Process_2021”, and “Reactome_2022” libraries. Visualization was performed using the seaborn library (v. 0.12.2) in python3 (v. 3.9.7). In addition, the ProteinAtlas database (https://www.proteinatlas.org/, v. 23.0, accessed on 10 October 2024) was used to interpret the data.

## 5. Conclusions

In the context of the effective treatment of CRC and LC, the primary task is to find approaches that make use of minimally invasive diagnostics. Using the MS profiling of secretome samples and EVs isolated from the culture medium of model CRC cell lines (Caco-2, HT29, and HCT116) and LC cell lines (A549, NCI-H23, and NCI-H460), we were able to identify dozens of potential secreted markers of CRC and LC. These highly biologically significant proteins play an important role in metastasis and tumor progression. These proteins may also be key players in the regulation of metabolic processes, including the response to oxidative stress, and may be involved in protein folding and telomere organization. Interestingly, many of the secreted markers identified in this study were previously considered as tissue markers of LC/CRC [33,35,49]. However, the main method used for studying their expression at the protein level was immunohistochemistry, which could potentially not be applicable for either multiplexed research or analysis in human blood. Using the method of targeted MS with isotope-labeled peptide standards (SRM/SIS), we have measured a number of EV-associated proteins, including SERPINA1 and CST3, that were determined to be present in model cell lines of CRC and in EVs isolated from the blood of patients with CRC and healthy volunteers. In the future, we plan to create an expanded SRM/SIS method based on the above results and use it for the analysis of tumor-secreted proteins in whole blood human plasma (HPL) and HPL-isolated EVs obtained from patients with CRC and LC.

## Figures and Tables

**Table 1:** Characteristics of patients with CRC and healthy volunteers.

	CRC	Healthy Volunteers
Total	20	19
Age	51–65	45–74
Male	10	10
Female	10	9
Stage 2 (T2N0M0)	20	-

## Data Availability

The mass spectrometry proteomics data have been deposited in the ProteomeXchange Consortium via the PRIDE partner repository with the dataset identifier PXD057205. Dataset is available via link https://www.ebi.ac.uk/pride/archive/projects/PXD057205/privatereviewdataset (accessed on 9 January 2025) with dataset identifier PXD057205 and token xEGottuKcC74.

## Supplementary Materials

The following supporting information can be downloaded at: https://www.mdpi.com/article/10.3390/ijms26031016/s1.

## References

[1] Rawla P., Sunkara T., Barsouk A. (2019). Epidemiology of Colorectal Cancer: Incidence, Mortality, Survival, and Risk Factors. Przegląd Gastroenterol..

[2] Eng C., Yoshino T., Ruíz-García E., Mostafa N., Cann C.G., O’Brian B., Benny A., Perez R.O., Cremolini C. (2024). Colorectal Cancer. Lancet.

[3] Araghi M., Mannani R., Heidarnejad maleki A., Hamidi A., Rostami S., Safa S.H., Faramarzi F., Khorasani S., Alimohammadi M., Tahmasebi S. (2023). Recent Advances in Non-Small Cell Lung Cancer Targeted Therapy; an Update Review. Cancer Cell Int..

[4] Jeon D.S., Kim H.C., Kim S.H., Kim T.-J., Kim H.K., Moon M.H., Beck K.S., Suh Y.-G., Song C., Ahn J.S. (2023). Five-Year Overall Survival and Prognostic Factors in Patients with Lung Cancer: Results from the Korean Association of Lung Cancer Registry (KALC-R) 2015. Cancer Res. Treat..

[5] Huck M.B., Bohl J.L. (2016). Colonic Polyps: Diagnosis and Surveillance. Clin. Colon Rectal Surg..

[6] Tran T.N., Bouchat J., Peeters M., Berghmans B., Van Cutsem E., Van Hal G., Van Herck K., Hoeck S. (2024). Bleeding and Perforation Complications after Follow-Up Colonoscopies in Faecal Immunochemical Test-Based Colorectal Cancer Screening: Insights from a Retrospective Case–Control Study. Gastrointest. Disord..

[7] Meklin J., Syrjänen K., Eskelinen M. (2020). Colorectal Cancer Screening with Traditional and New-Generation Fecal Immunochemical Tests: A Critical Review of Fecal Occult Blood Tests. Anticancer Res..

[8] Hauptman N., Glavač D. (2017). Colorectal Cancer Blood-Based Biomarkers. Gastroenterol. Res. Pract..

[9] Thai A.A., Solomon B.J., Sequist L.V., Gainor J.F., Heist R.S. (2021). Lung Cancer. Lancet.

[10] Pinsky P.F. (2014). Assessing the Benefits and Harms of Low-Dose Computed Tomography Screening for Lung Cancer. Lung Cancer Manag..

[11] Romero D. (2020). NELSON Updated. Nat. Rev. Clin. Oncol..

[12] Aberle D.R., Adams A.M., Berg C.D., Black W.C., Clapp J.D., Fagerstrom R.M., Gareen I.F., Gatsonis C., Marcus P.M., Sicks J.D. (2011). Reduced Lung-Cancer Mortality with Low-Dose Computed Tomographic Screening. N. Engl. J. Med..

[13] Forsberg A., Westerberg M., Metcalfe C., Steele R., Blom J., Engstrand L., Fritzell K., Hellström M., Levin L.-Å., Löwbeer C. (2022). Once-Only Colonoscopy or Two Rounds of Faecal Immunochemical Testing 2 Years Apart for Colorectal Cancer Screening (SCREESCO): Preliminary Report of a Randomised Controlled Trial. Lancet Gastroenterol. Hepatol..

[14] Hossain M.S., Karuniawati H., Jairoun A.A., Urbi Z., Ooi D.J., John A., Lim Y.C., Kibria K.M.K., Mohiuddin A.K.M., Ming L.C. (2022). Colorectal Cancer: A Review of Carcinogenesis, Global Epidemiology, Current Challenges, Risk Factors, Preventive and Treatment Strategies. Cancers.

[15] Olmedo M.E., Cervera R., Cabezon-Gutierrez L., Lage Y., Corral de la Fuente E., Gómez Rueda A., Mielgo-Rubio X., Trujillo J.C., Couñago F. (2022). New Horizons for Uncommon Mutations in Non-Small Cell Lung Cancer: BRAF, KRAS, RET, MET, NTRK, HER2. World J. Clin. Oncol..

[16] Dang X.T.T., Kavishka J.M., Zhang D.X., Pirisinu M., Le M.T.N. (2020). Extracellular Vesicles as an Efficient and Versatile System for Drug Delivery. Cells.

[17] Ponomarenko E.A., Poverennaya E.V., Ilgisonis E.V., Pyatnitskiy M.A., Kopylov A.T., Zgoda V.G., Lisitsa A.V., Archakov A.I. (2016). The Size of the Human Proteome: The Width and Depth. Int. J. Anal. Chem..

[18] Anderson N.L., Anderson N.G. (2002). The Human Plasma Proteome: History, Character, and Diagnostic Prospects. Mol. Cell. Proteom..

[19] Harlid S., Gunter M.J., Van Guelpen B. (2021). Risk-Predictive and Diagnostic Biomarkers for Colorectal Cancer; a Systematic Review of Studies Using Pre-Diagnostic Blood Samples Collected in Prospective Cohorts and Screening Settings. Cancers.

[20] El-Khoury V., Schritz A., Kim S.-Y., Lesur A., Sertamo K., Bernardin F., Petritis K., Pirrotte P., Selinsky C., Whiteaker J.R. (2020). Identification of a Blood-Based Protein Biomarker Panel for Lung Cancer Detection. Cancers.

[21] Ignjatovic V., Geyer P.E., Palaniappan K.K., Chaaban J.E., Omenn G.S., Baker M.S., Deutsch E.W., Schwenk J.M. (2019). Mass Spectrometry-Based Plasma Proteomics: Considerations from Sample Collection to Achieving Translational Data. J. Proteome Res..

[22] Soloveva N., Novikova S., Farafonova T., Tikhonova O., Zgoda V. (2023). Proteomic Signature of Extracellular Vesicles Associated with Colorectal Cancer. Molecules.

[23] Novikova S.E., Soloveva N.A., Farafonova T.E., Tikhonova O.V., Liao P.-C., Zgoda V.G. (2021). Proteomic Signature of Extracellular Vesicles for Lung Cancer Recognition. Molecules.

[24] Novikova S., Shushkova N., Farafonova T., Tikhonova O., Kamyshinsky R., Zgoda V. (2020). Proteomic Approach for Searching for Universal, Tissue-Specific, and Line-Specific Markers of Extracellular Vesicles in Lung and Colorectal Adenocarcinoma Cell Lines. Int. J. Mol. Sci..

[25] Yuan X.-H., Yang J., Wang X.-Y., Zhang X.-L., Qin T.-T., Li K. (2018). Association between EGFR/KRAS Mutation and Expression of VEGFA, VEGFR and VEGFR2 in Lung Adenocarcinoma. Oncol. Lett..

[26] Alshangiti A., Chandhoke G., Ellis P.M. (2018). Antiangiogenic Therapies in Non-Small-Cell Lung Cancer. Curr. Oncol..

[27] Sirvent A., Espie K., Papadopoulou E., Naim D., Roche S. (2022). New Functions of DDR1 Collagen Receptor in Tumor Dormancy, Immune Exclusion and Therapeutic Resistance. Front. Oncol..

[28] Jing H., Song J., Zheng J. (2018). Discoidin Domain Receptor 1: New Star in Cancer-Targeted Therapy and Its Complex Role in Breast Carcinoma. Oncol. Lett..

[29] Sirvent A., Lafitte M., Roche S. (2018). DDR1 Inhibition as a New Therapeutic Strategy for Colorectal Cancer. Mol. Cell. Oncol..

[30] Tao Y., Wang R., Lai Q., Wu M., Wang Y., Jiang X., Zeng L., Zhou S., Li Z., Yang T. (2019). Targeting of DDR1 with Antibody-Drug Conjugates Has Antitumor Effects in a Mouse Model of Colon Carcinoma. Mol. Oncol..

[31] Gu Y., Ding X., Huang J., Xue M., Zhang J., Wang Q., Yu H., Wang Y., Zhao F., Wang H. (2018). The Deubiquitinating Enzyme UCHL1 Negatively Regulates the Immunosuppressive Capacity and Survival of Multipotent Mesenchymal Stromal Cells. Cell Death Dis..

[32] Luo Y., He J., Yang C., Orange M., Ren X., Blair N., Tan T., Yang J.-M., Zhu H. (2018). UCH-L1 Promotes Invasion of Breast Cancer Cells through Activating Akt Signaling Pathway. J. Cell. Biochem..

[33] Goto Y., Zeng L., Yeom C.J., Zhu Y., Morinibu A., Shinomiya K., Kobayashi M., Hirota K., Itasaka S., Yoshimura M. (2015). UCHL1 Provides Diagnostic and Antimetastatic Strategies Due to Its Deubiquitinating Effect on HIF-1α. Nat. Commun..

[34] Carolan B.J., Heguy A., Harvey B.-G., Leopold P.L., Ferris B., Crystal R.G. (2006). Up-Regulation of Expression of the Ubiquitin Carboxyl-Terminal Hydrolase L1 Gene in Human Airway Epithelium of Cigarette Smokers. Cancer Res..

[35] Yao J., Reyimu A., Sun A., Duoji Z., Zhou W., Liang S., Hu S., Wang X., Dai J., Xu X. (2022). UCHL1 Acts as a Potential Oncogene and Affects Sensitivity of Common Anti-Tumor Drugs in Lung Adenocarcinoma. World J. Surg. Oncol..

[36] Tang X., Jin R., Qu G., Wang X., Li Z., Yuan Z., Zhao C., Siwko S., Shi T., Wang P. (2013). GPR116, an Adhesion G-Protein-Coupled Receptor, Promotes Breast Cancer Metastasis via the Gαq-P63RhoGEF-Rho GTPase Pathway. Cancer Res..

[37] Zheng T., Sun M., Liu L., Lan Y., Wang L., Lin F. (2021). GPR116 Overexpression Correlates with Poor Prognosis in Gastric Cancer. Medicine.

[38] De Masi R., Orlando S. (2022). GANAB and N-Glycans Substrates Are Relevant in Human Physiology, Polycystic Pathology and Multiple Sclerosis: A Review. Int. J. Mol. Sci..

[39] Thomas D., Rathinavel A.K., Radhakrishnan P. (2021). Altered Glycosylation in Cancer: A Promising Target for Biomarkers and Therapeutics. Biochim. Biophys. Acta Rev. Cancer.

[40] Zhang Y., Qian C., Jing L., Ren J., Guan Y. (2017). Meta-Analysis Indicating That High ALCAM Expression Predicts Poor Prognosis in Colorectal Cancer. Oncotarget.

[41] Wang M., Qu L., Du X., Song P., Ng J.P.L., Wong V.K.W., Law B.Y.K., Fu X. (2024). Natural Products and Derivatives Targeting Metabolic Reprogramming in Colorectal Cancer: A Comprehensive Review. Metabolites.

[42] Wang M., Zhang H., Lu Z., Su W., Tan Y., Wang J., Jia X. (2024). PSAT1 Mediated EMT of Colorectal Cancer Cells by Regulating Pl3K/AKT Signaling Pathway. J. Cancer.

[43] Zhang J., Zou S., Fang L. (2023). Metabolic Reprogramming in Colorectal Cancer: Regulatory Networks and Therapy. Cell Biosci..

[44] Osumi H., Shinozaki E., Osako M., Kawazoe Y., Oba M., Misaka T., Goto T., Kamo H., Suenaga M., Kumekawa Y. (2015). Cetuximab Treatment for Metastatic Colorectal Cancer with KRAS p.G13D Mutations Improves Progression-Free Survival. Mol. Clin. Oncol..

[45] Hu H., Gong B., Zhang M. (2020). The Expression of Apolipoproteina1 and Its Correlation with Infiltration of Urologic Neoplasm. Transl. Cancer Res..

[46] He Y., Chen J., Ma Y., Chen H. (2022). Apolipoproteins: New Players in Cancers. Front. Pharmacol..

[47] Nikitidou E., Khoonsari P.E., Shevchenko G., Ingelsson M., Kultima K., Erlandsson A. (2017). Increased Release of Apolipoprotein E in Extracellular Vesicles Following Amyloid-β Protofibril Exposure of Neuroglial Co-Cultures. J. Alzheimers Dis..

[48] Jiang S., Li X., Li Y., Chang Z., Yuan M., Zhang Y., Zhu H., Xiu Y., Cong H., Yin L. (2024). APOE from Patient-Derived Astrocytic Extracellular Vesicles Alleviates Neuromyelitis Optica Spectrum Disorder in a Mouse Model. Sci. Transl. Med..

[49] He L., Shi M., Ren S., Zhang J., Tian Y., Yang X., Liu H. (2023). Jun-APOE-LRP1 Axis Promotes Tumor Metastasis in Colorectal Cancer. Biomol. Biomed..

[50] Ruggeri J.M., Franco-Barraza J., Sohail A., Zhang Y., Long D., Pasca di Magliano M., Cukierman E., Fridman R., Crawford H.C. (2020). Discoidin Domain Receptor 1 (DDR1) Is Necessary for Tissue Homeostasis in Pancreatic Injury and Pathogenesis of Pancreatic Ductal Adenocarcinoma. Am. J. Pathol..

[51] Wilson C.L., Murphy L.B., Leslie J., Kendrick S., French J., Fox C.R., Sheerin N.S., Fisher A., Robinson J.H., Tiniakos D.G. (2015). Ubiquitin C-Terminal Hydrolase 1: A Novel Functional Marker for Liver Myofibroblasts and a Therapeutic Target in Chronic Liver Disease. J. Hepatol..

[52] Bari E., Ferrarotti I., Di Silvestre D., Grisoli P., Barzon V., Balderacchi A., Torre M.L., Rossi R., Mauri P., Corsico A.G. (2019). Adipose Mesenchymal Extracellular Vesicles as Alpha-1-Antitrypsin Physiological Delivery Systems for Lung Regeneration. Cells.

[53] Soloveva N.A., Novikova S.E., Farafonova T.E., Tikhonova O.V., Zgoda V.G., Archakov A.I. (2024). Proteome of Plasma Extracellular Vesicles as a Source of Colorectal Cancer Biomarkers. Biomed. Khim..

[54] Oh H.S.-H., Rutledge J., Nachun D., Pálovics R., Abiose O., Moran-Losada P., Channappa D., Urey D.Y., Kim K., Sung Y.J. (2023). Organ Aging Signatures in the Plasma Proteome Track Health and Disease. Nature.

[55] Wessel D., Flügge U.I. (1984). A Method for the Quantitative Recovery of Protein in Dilute Solution in the Presence of Detergents and Lipids. Anal. Biochem..

[56] Wiśniewski J.R., Zougman A., Nagaraj N., Mann M. (2009). Universal Sample Preparation Method for Proteome Analysis. Nat. Methods.

