# Supplementary material for: Secretome and Proteome of Extracellular Vesicles Provide Protein Markers of Lung and Colorectal Cancer
Source: Int J Mol Sci. 2025 Jan 25;26(3):1016. doi: 10.3390/ijms26031016 (PMC11816676; doi:10.3390/ijms26031016)
Supplement: Supplementary file 1 [file ijms-26-01016-s001.zip › Supplementary materials.pdf]

Supplementary materials

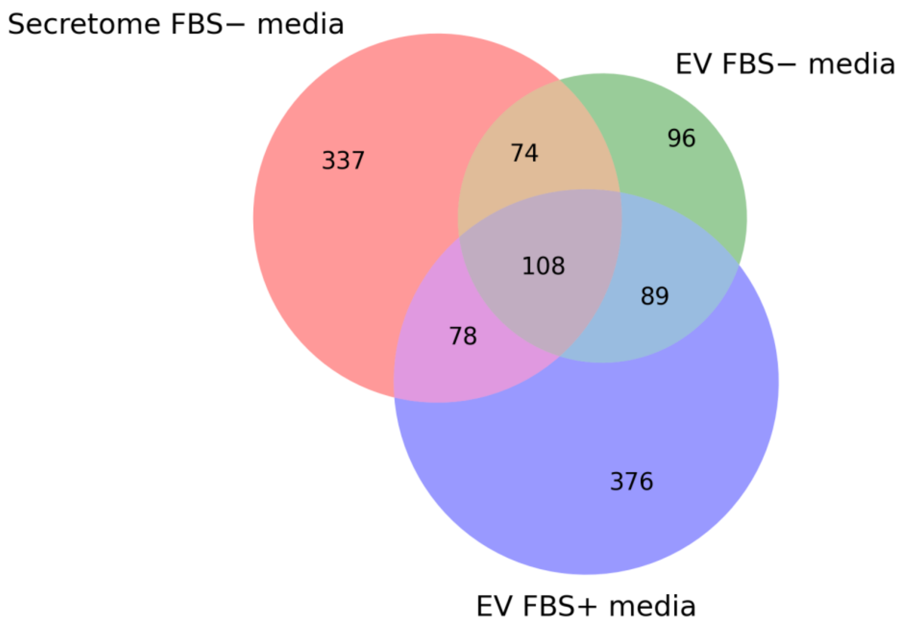

Figure S1. Venn diagram demonstrating the intersection between proteins confidently identified by at least two unique peptides in secretome and EV samples, which were derived from FBS-free media (FBS -) in current study, and EV samples, which were derived from FBS-supplemented media (FBS +) in our previous work [24].

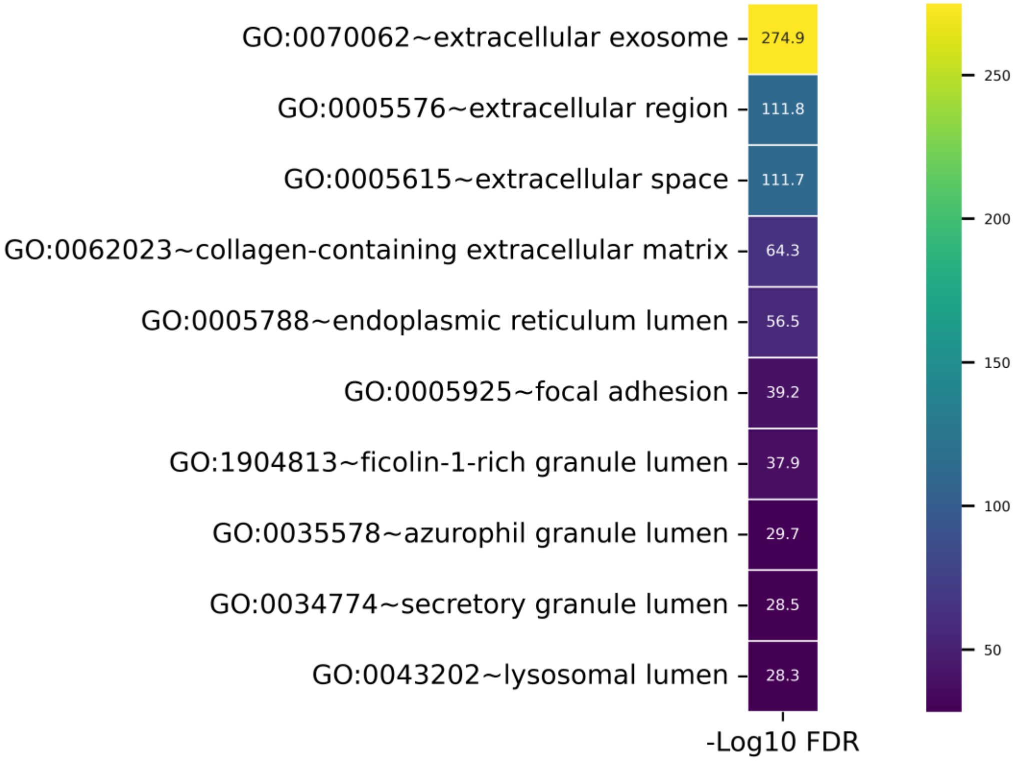

Figure S2. Annotation of the cellular localization and biological significance of proteins detected in secretome and EV samples for all cell lines studied (N = 782, identified by at least two unique peptides).

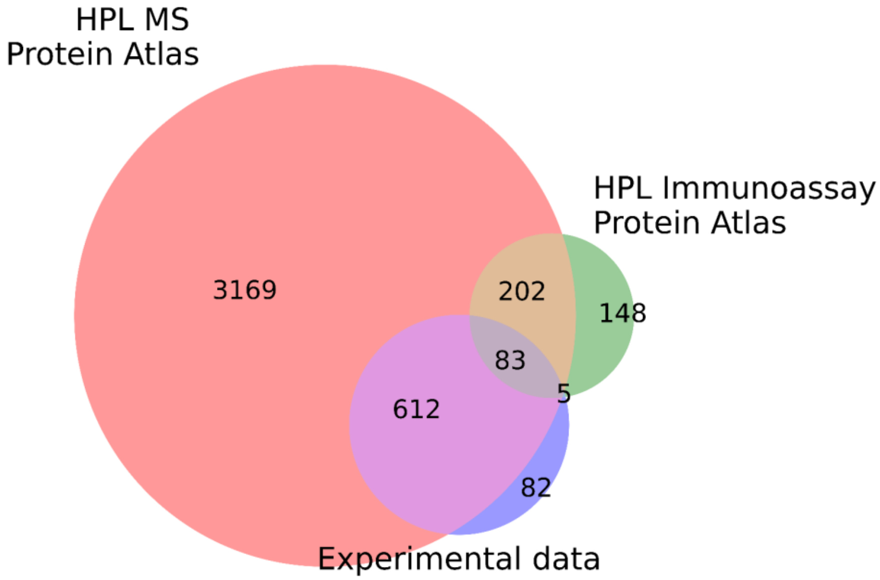

Figure S3. Venn diagram showing the intersection of experimental data with a list of proteins from the Protein Atlas database (v. 23.0, last accessed 10-10-2024). HPL MS - proteins detected in plasma using mass spectrometric measurements Protein Atlas (v. 23.0, last accessed 10-10-2024), HPL Immunoassay - proteins detected in plasma using immunoassay, Protein Atlas (v. 23.0, last accessed 10-10-2024).

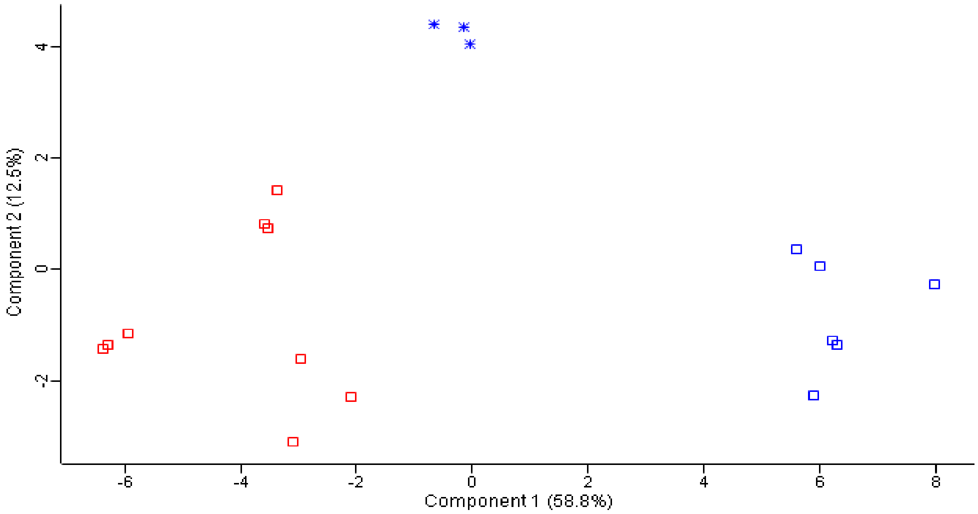

Figure S4. Principal component analysis of the secretome proteins of CRC (blue) and LC (red) cell lines. Asterisks indicate the result for the CRC cell line HCT116. Visualization performed in Perseus 1.6.0.7 software (Max Planck Institute of Biochemistry, Martinsried, Germany).

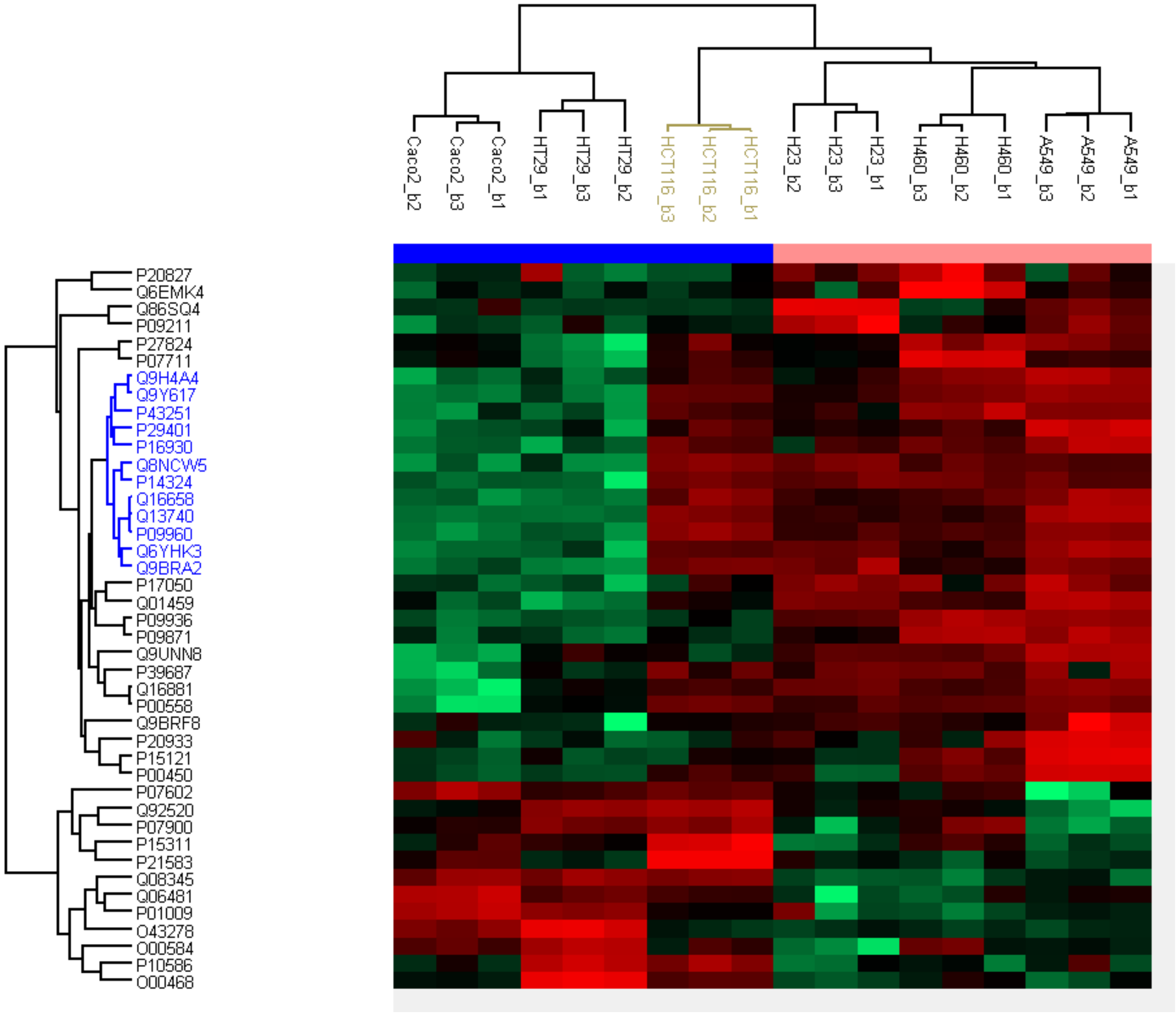

Figure S5. Heatmap of 42 secretome proteins in CRC (blue columns) and LC (pink columns). Rows highlighted in blue indicate a cluster of CRC secretome proteins that are expressed at levels similar to those in LC cell lines. Visualization performed in Perseus 1.6.0.7 software (Max Planck Institute of Biochemistry, Martinsried, Germany).

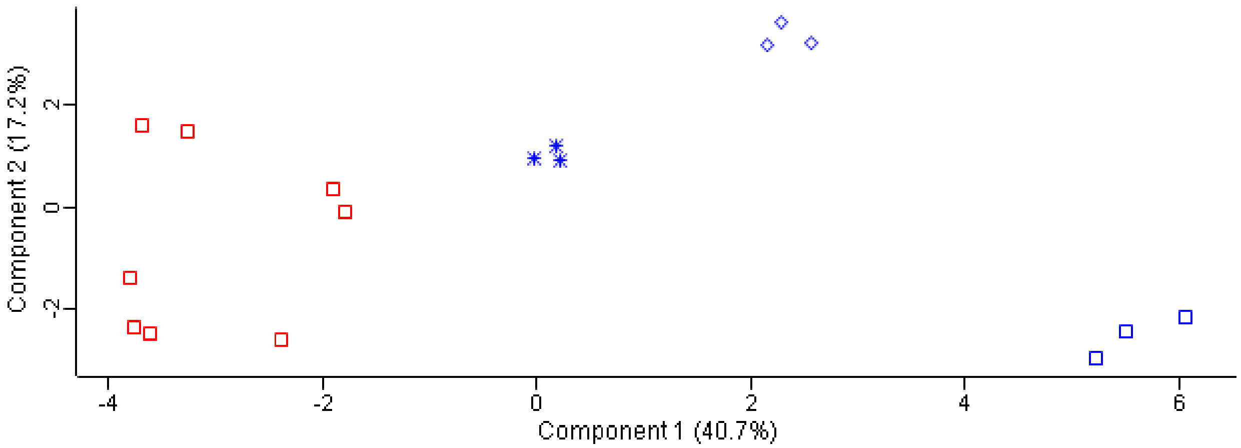

Figure S6. Principal component analysis of the EV proteins of the CRC (blue) and LC (red) lines. Asterisks indicate the result for the CRC cell line HCT116. Visualization performed in Perseus 1.6.0.7 software (Max Planck Institute of Biochemistry, Martinsried, Germany).

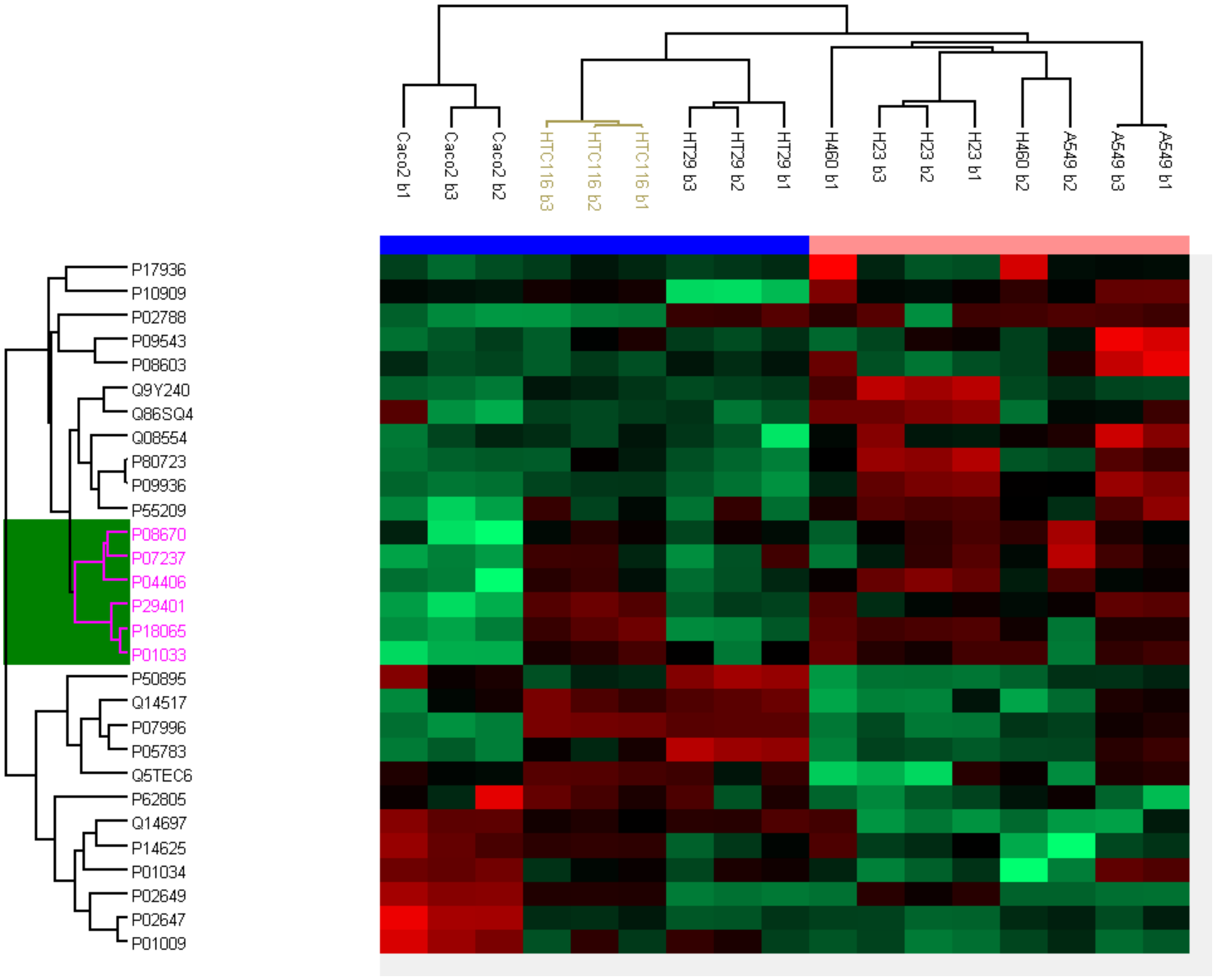

Figure S7. Heatmap of 29 proteins from EV samples derived from CRC cell lines (blue columns) and LC cell lines (pink columns). Rows highlighted in blue indicate a cluster of proteins in CRC cells that are expressed at levels similar to those in LC cell lines. Visualization performed in Perseus 1.6.0.7 software (Max Planck Institute of Biochemistry, Martinsried, Germany).

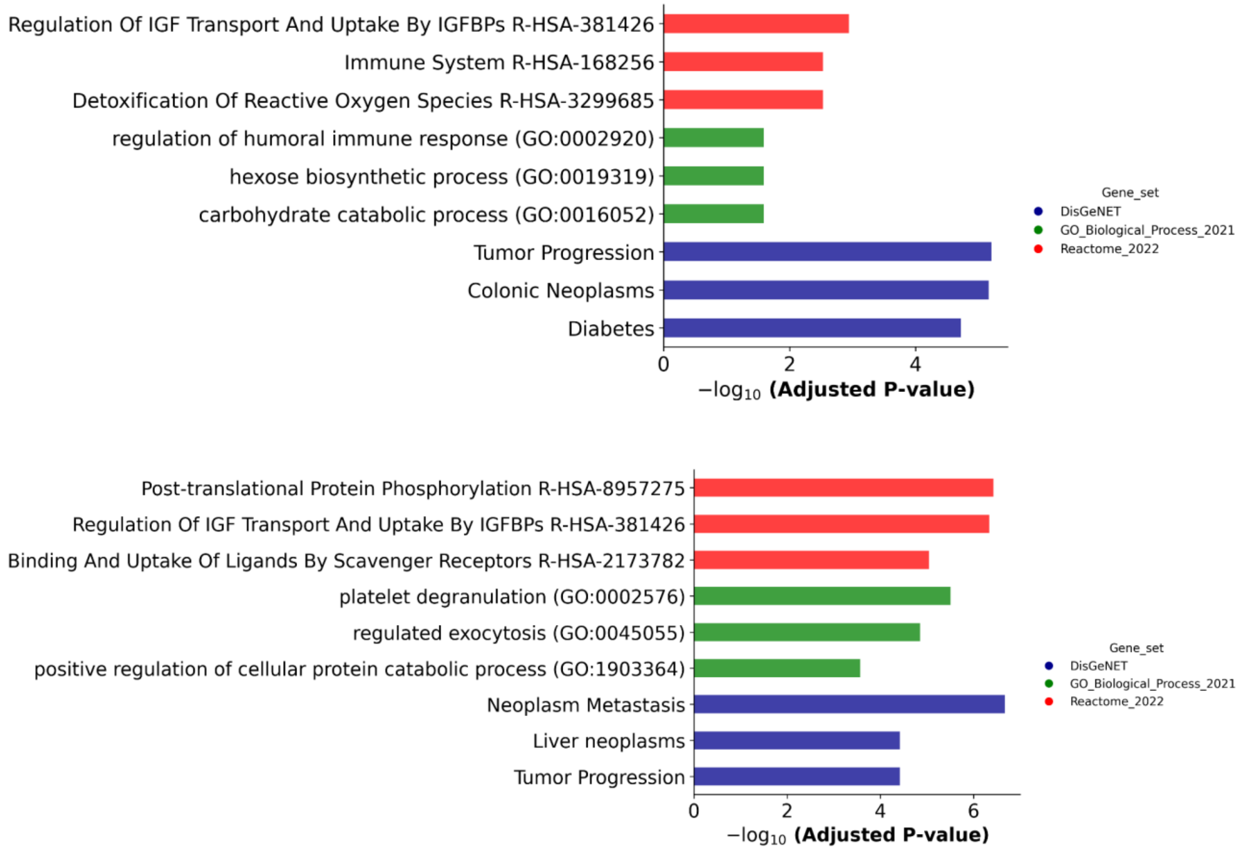

Figure S8. Result of enrichment analysis for 23 secreted CRC markers (upper panel) and 44 secreted LC markers (lower panel) performed with gseapy (v. 1.0.4) against DisGenNET, Reactome, and GO databases by Biological Process categories, adjusted P-value <0.05, showing the top three most significant groups from each resource. Enrichment analysis was performed with gseapy (v. 1.0.4) in python3 (v. 3.9.7) against "DisGenNET", "GO_Biological_Process_2021", and "Reactome_2022" libraries.

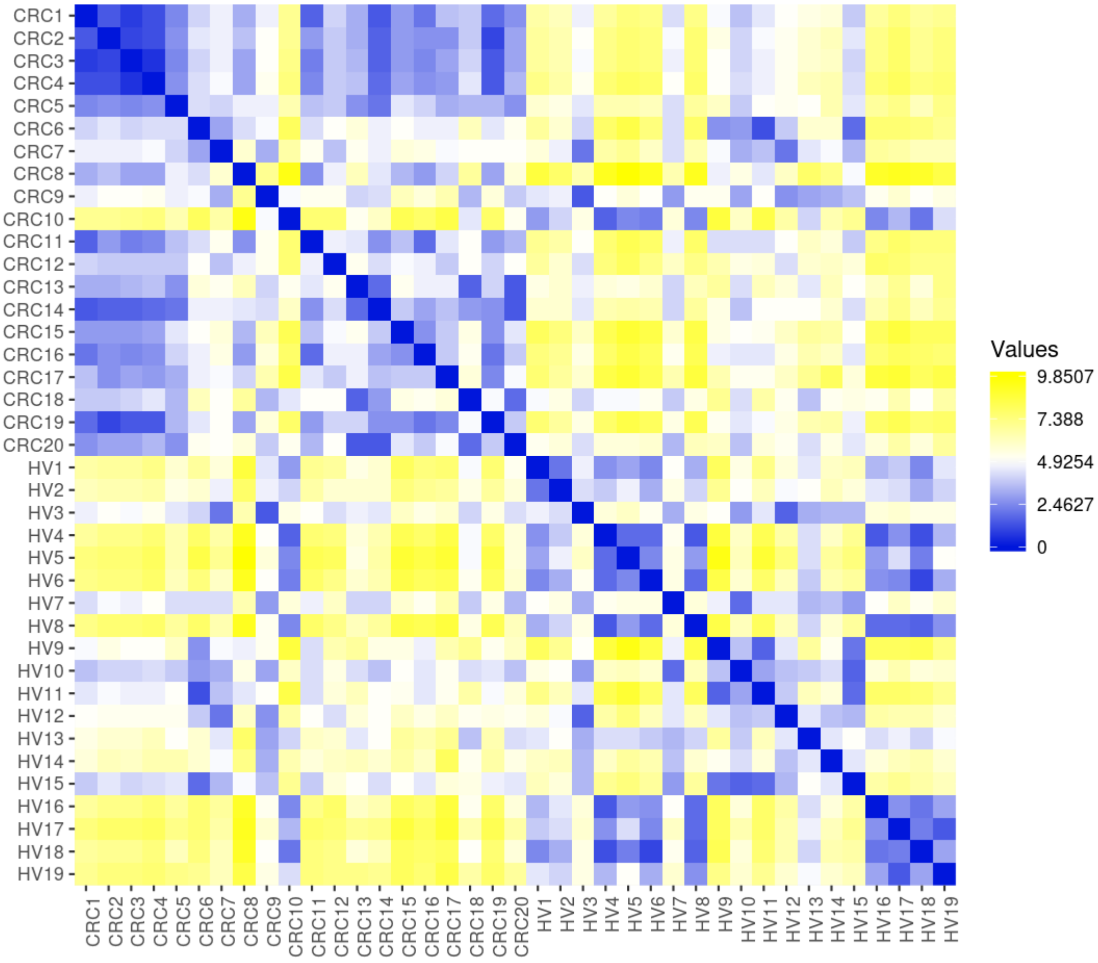

Figure S9. Distance matrix constructed based on the expression of 11 proteins in EV samples isolated from blood plasma of CRC patients (CRC), and healthy volunteers (HV). Obtained by Heatmapper (http://www.heatmapper.ca, last accessed 10-10-2024)

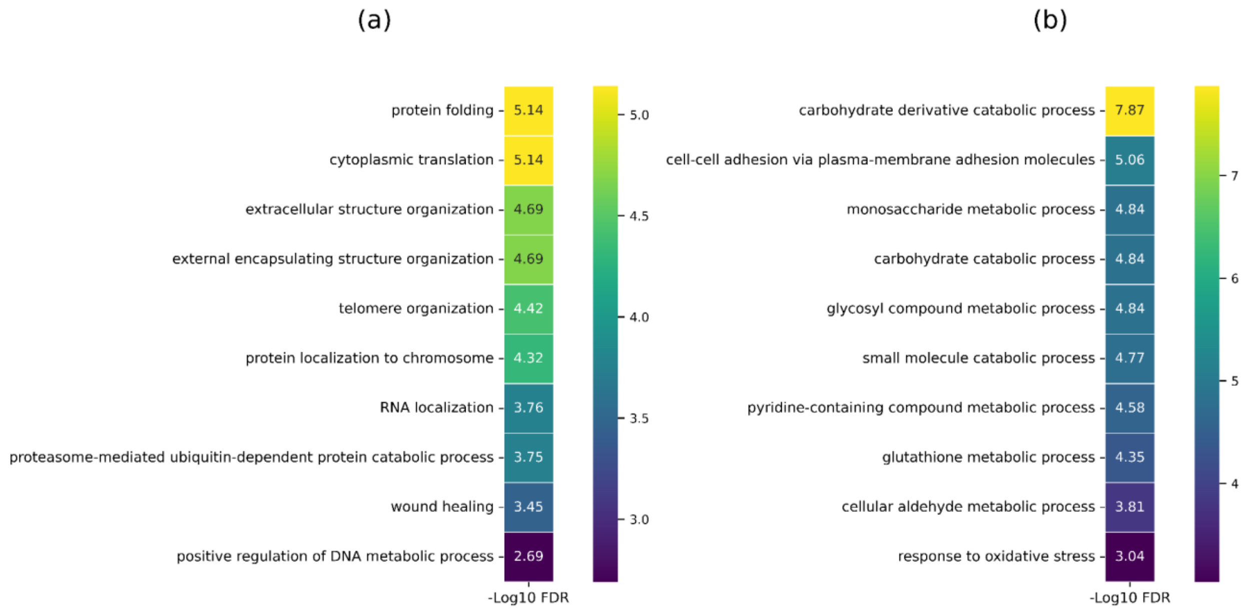

Figure S1. Functional annotation of proteins detected only in the EV proteome samples (a) or only in the secretome samples (b) from LC and CRC cells. Enrichment analysis was performed using the WebGestalt online resource (https://www.webgestalt.org/, last accessed 08-10-2024) against the GO database categories "geneontology_Biological_Process_noRedundant" (FDR Method: BH). The statistically most significant groups of biological processes are presented. Visualization was performed using the seaborn library (v. 0.12.2) in python3 (v. 3.9.7).

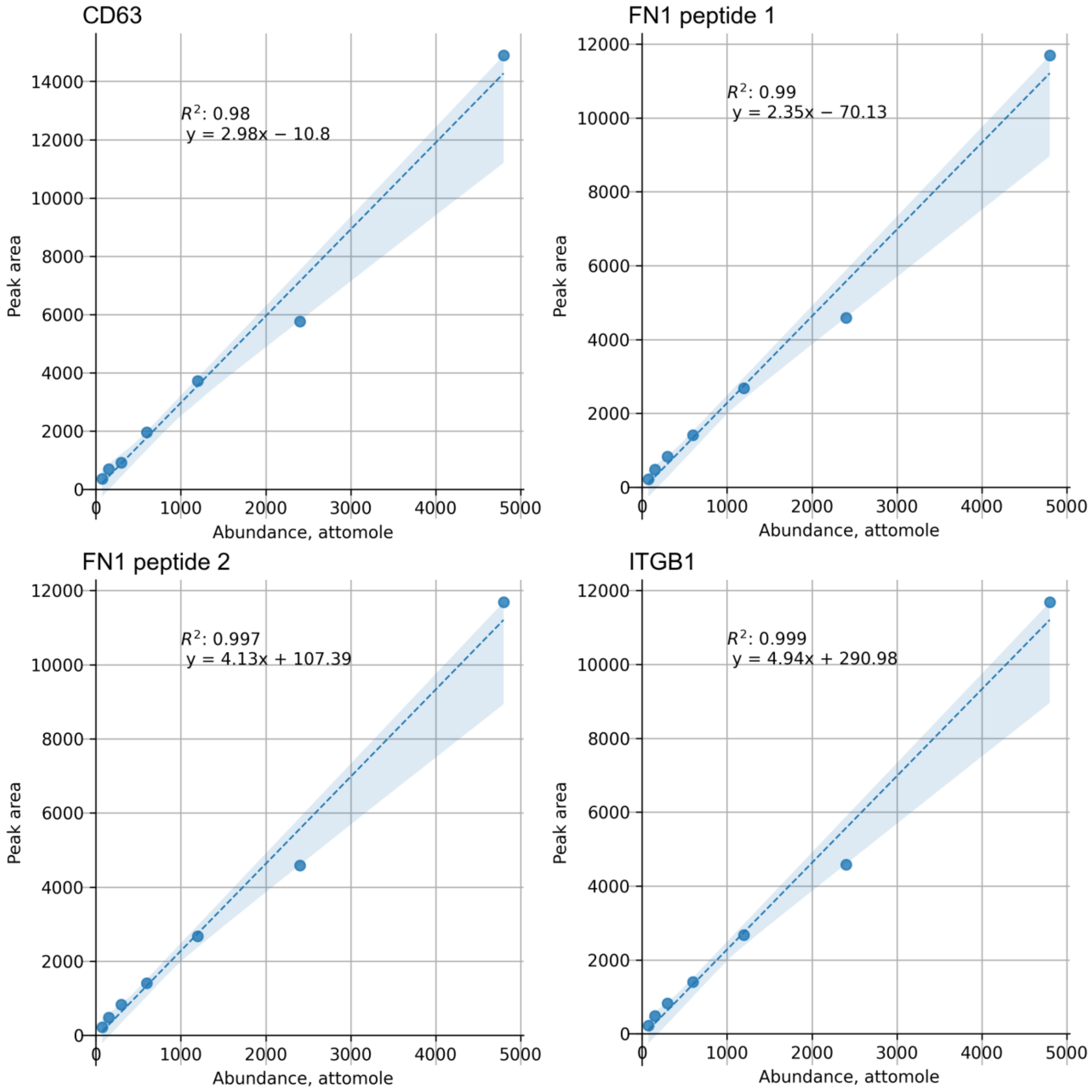

Figure S21. Calibration curves for SIS peptides VMSEFNNNFR (mapped on CD63 protein, LOD/LOQ = 66/202 attomole), SYTITGLQPGTDYK (peptide 1 mapped on FN1, LOD/LOQ = 35/106 attomole), STTPDITGYR (peptide 2 mapped on FN1, LOD/LOQ = 75/227 attomole), and SAVTTVVNPK (mapped on ITGB1 protein, LOD/LOQ = 17/51 attomole). the x-axis shows SIS abundance per LC/SRM injection; the y-axis shows peak area under extracted ion chromatogram obtained by SRM analysis, SIS peptides were measured at the levels of 75, 150, 300, 600,1200, 2400, 4800 attomole, diluted in the presence of non-targeted peptide matrix. The LOD and LOQ were determined based on the slope of the calibration curve and three and ten standard deviations of the response, respectively.

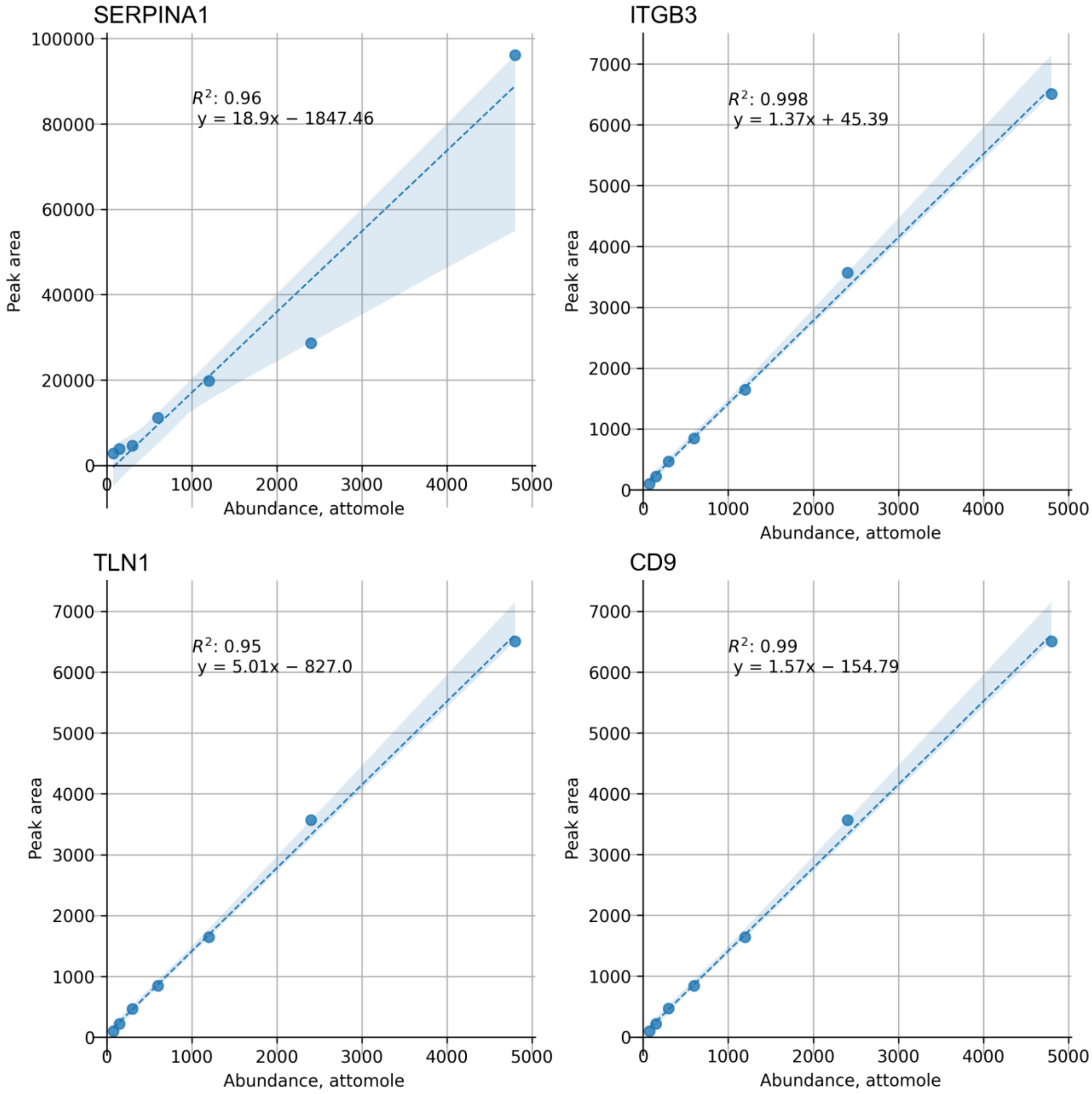

Figure S13. Calibration curves for SIS peptides LSITGTYDLK (mapped on SERPINA1 protein, LOD/LOQ = 804/2437 attomole), HVLTLTDQVTR (mapped on ITGB3, LOD/LOQ = 27/80 attomole), GLAGAVSELLR (mapped on TLN1, LOD/LOQ = 24/74 attomole), and DVLETFTVK (mapped on CD9 protein, LOD/LOQ = 21/64 attomole). the x-axis shows SIS abundance per LC/SRM injection; the y-axis shows peak area under extracted ion chromatogram obtained by SRM analysis, SIS peptides were measured at the levels of 75, 150, 300, 600,1200, 2400, 4800 attomole, diluted in the presence of non-targeted peptide matrix. The LOD and LOQ were determined based on the slope of the calibration curve and three and ten standard deviations of the response, respectively.

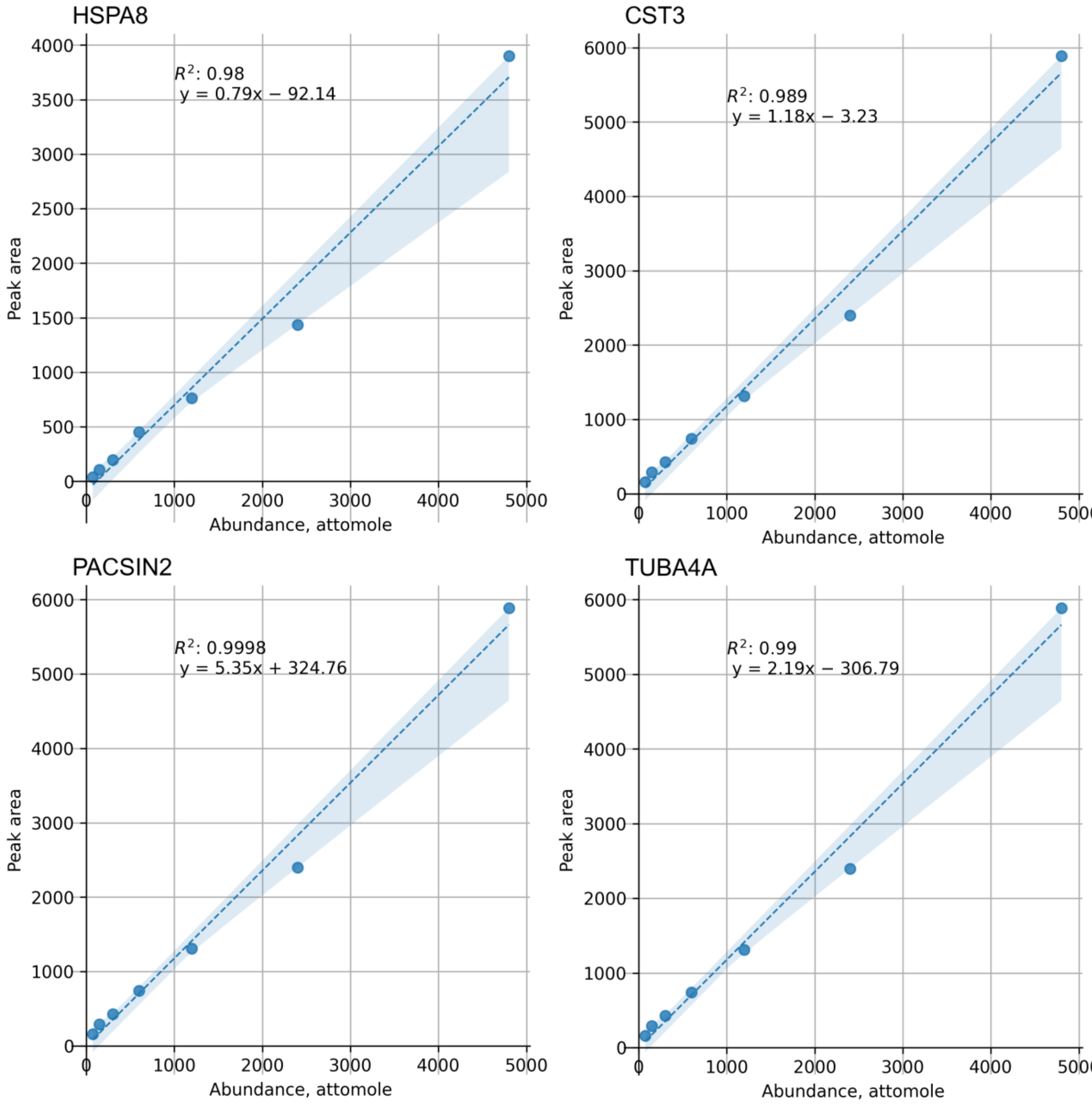

Figure S14. Calibration curves for SIS peptides DAGTIAGLNVLR (mapped on HSPA8 protein, LOD/LOQ = 42/126 attomole), ALDFAVGEYNK (mapped on CST3, LOD/LOQ = 14/42 attomole), AADAVEDLR (mapped on PACSIN2, LOD/LOQ = 13/39 attomole), and AVFVDLEPTVIDEIR (mapped on TUBA4A protein, LOD/LOQ = 14/45 attomole). the x-axis shows SIS abundance per LC/SRM injection; the y-axis shows peak area under extracted ion chromatogram obtained by SRM analysis, SIS peptides were measured at the levels of 75, 150, 300, 600,1200, 2400, 4800 attomole, diluted in the presence of non-targeted peptide matrix. The LOD and LOQ were determined based on the slope of the calibration curve and three and ten standard deviations of the response, respectively.

Table S2. Cell lines features

Cell Line	Tissue	Histology	Mutated genes
A549	Lung, epithelial cell	Non small cell lung carcinoma	KRAS, STK11, TP53
NCI-H23	Lung	Lung adenocarcinom a	ATM, KRAS, STK11, TP53
NCI-H460	Lung	non-small cell lung cancer	KRAS, PIK3CA, STK11, TP53
HCT116	Large intestine; Colon, epithelial cell	Carcinoma; Colorectal	ACVR2A, BRCA2, CDKN2A, EP300, KRAS, PIK3CA, PPM1D, TGFBR2, TP53
Caco2	Large intestine; Colon, epithelial cell	Colorectal Adenocarcino ma	APC, CTNNB1, SMAD4, TP53
HT-29	Colon, epithelial cell	Colorectal Adenocarcino ma	APC, BRAF, PIK3CA, SMAD4, TP53
